# Reduction of silver ions in molybdates: elucidation of framework acidity as the factor controlling charge balance mechanisms in aqueous zinc-ion electrolyte†

**DOI:** 10.1039/d1ra07765a

**Published:** 2021-12-13

**Authors:** Derrick Combs, Brendan Godsel, Julie Pohlman-Zordan, Allen Huff, Jackson King, Robert Richter, Paul F. Smith

**Affiliations:** Department of Chemistry, Valparaiso University 1710 Chapel Drive Valparaiso IN 46383 USA paul.smith1@valpo.edu; Department of Chemistry and Physics, Chicago State University 9501 S. King Drive Chicago IL 60628 USA

## Abstract

A percolating network of high electrical conductivity needed to operate electrodes at a fast rate can be formed by *in situ* reduction of Ag^+^ originating from mixed metal oxide lattices, but few studies have elucidated trends in this mechanism as a function of Ag^+^ concentration and structure. Candidates compared for the first time here are spinel Ag_2_MoO_4_, monoclinic and triclinic Ag_2_Mo_2_O_7_, and Ag_2_Mo_3_O_10_·2H_2_O, which have reduction potentials for Ag^+^ and Mo^6+^ strongly decoupled by up to ∼600 mV in aqueous zinc-ion electrolyte. Under these conditions, Ag^0^ is the first reduction product and a decrease of charge transfer resistance by ∼100× is observed within 2.5% consumption of total Ag^+^ independent of initial structure. However, resistance metrics alone poorly describe materials which are robust to reducing silver with high energy at faster rates. Instead, after accounting for crystallinity and morphology differences, we find that the acidity of the molybdate framework is responsible for a switch in charge balance mechanism from the bulk formation of a mixed ZnMoO_*x*_ to pseudocapacitive Zn^2+^ precipitation, and that this mechanism switch is associated with minimized losses to rate, voltage and capacity yields as carbon/binder free electrodes relative to composites. The location of this acidity cutoff near the pH of the ZnSO_4_ electrolyte may suggest a design principle for future low-carbon electrodes beyond molybdate framework structures.

## Abstract 2

Across four molybdates, reduction of silver ions in aqueous zinc electrolyte is more facile with increasing acidity.

## Graphical abstract

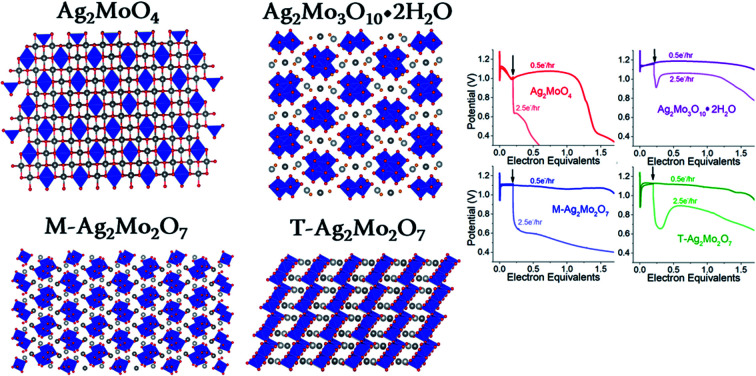

## Introduction

Electrodes must increasingly support faster electron transport as the devices they power become more demanding. Towards this goal, the development of materials containing Ag^+^ is pursued for fundamental interest because the reduced product can contain a network of atomically dispersed, conductive Ag^0^ particles that support fast electron transport throughout the bulk.^1,2^ Minute amounts of dispersed Ag^+^ are known to significantly improve electronic transport; as example, cathodes of the silver–vanadium–phosphate-oxide type show percolation thresholds with 0.3% volume reduction of Ag^+^ concurrently resulting in 10^6^ decrease in electrical resistance.^3–5^ Silver vanadium oxide (SVO) cathodes remain the dominant material in implantable cardiac defibrillator batteries due to this reductive displacement mechanism.^6^

Given that vanadium oxide cathodes have shown tremendous promise in aqueous zinc-ion batteries (AZIB's)^7–9^ recent efforts to incorporate reductive displacement cathodes in this new application have included Ag^0^-doped V_2_O_5_,^10^ Ag_0.4_V_2_O_5_,^11^ Ag_1.2_V_3_O_8_,^12^ Ag_2_V_4_O_11_,^13^ Ag_0.33_V_2_O_5_@V_2_O_5_·*n*H_2_O,^14^ β-AgVO_3_,^15^ and even Cu analogs in CuV_2_O_6_,^16,17^ Cu_0.95_V_2_O_5_,^18^ Cu_*x*_V_2_O_5_,^19^ Cu_0.34_V_2_O_5_ (ref. 20) and Cu_3_(OH)_2_V_2_O_7_.^21,22^ However, an early emphasis on reporting individual structural metrics – which we summarize in Table S1† – rather than critical comparisons has led to inconsistencies when viewed broadly that, to our knowledge, have not been addressed. Only one study attempts to directly compare any subset of these structures in AZIBs, finding tunneled Ag_0.33_V_2_O_5_ outperformed layered Ag_2_V_4_O_11_ and chain-like β-AgVO_3_ at cycle life, with the latter exhibiting rapid capacity fade to <25 mA h g^−1^ at 1000 mA g^−1^ rates in only 25 cycles.^12,23^ With reports that chain-like β-AgVO_3_ is capable of 95 mA h g^−1^ after 1000 cycles at 2000 mA g^−1^ rates with 78% retention^15^ and layered Ag_2_V_4_O_11_ is capable of 93% capacity retention after 6000 cycles at 5 A g^−1^ rates,^13^ it is clear there exist factors as yet unknown which either caused these materials to perform worse in the comparative study or caused them to improve individually. Among many possiblities,^24^ sample crystallinity and morphology may rank highly considering that the studies reporting improved metrics from β-AgVO_3_ and Ag_2_V_4_O_11_ prepared nanowires and the comparative test did not.

We argue that, irrespective of whether this conjecture is true, it is not needed if a new set of testing conditions is widely adopted for reductive displacement cathodes. After all, these are materials which, at their best, enable their own conductivity and should require electrodes with little (or no) additives, especially when compared to manufactured LiCoO_2_ and alkaline MnO_2_ cathodes with ∼86–95% active material.^25–27^ In spite of this tremendous advantage, all the aforementioned studies prepared electrodes with as much as 20% carbon and 10% binder. The consequence of this disconnect between the conditions in which SVO's should thrive *vs.* the conditions evaluated is that laudable metrics are reported irrespective of whether the SVO structure is tunneled, layered, or chain-like, and this fact is clearly the major contributor to the above inconsistency. For those in pursuit of structure/function relationships this should not be a satisfactory state of literature, especially considering the presence of Ag^+^ does not even guarantee reductive displacement occurs. For instance, lithiation of Ag_*x*_Mn_8_O_16_ (1.0 ≤ *x* ≤ 1.8) does not form Ag^0^ immediately, but rather Mn accepts the first ∼4 electron equivalents.^28,29^

Even with a lower reduction potential, V^5+^ is still competitive with Ag^+^ for early electrons in Ag_2_VO_2_PO_4_,^1^ resulting in Ag^0^ networks which may form differently as a function of rate^30^ and other factors,^31^ including the identity of the intercalating ion. Ag^0^ appears immediately when Ag_*x*_Mn_8_O_16_ is discharged in sodium batteries,^32^ and reoxidation of Ag^0^ which is not known in lithium batteries occurs in aqueous Zn systems.^33^ Clearly, there is a need to better understand reductive displacement mechanisms in AZIBs since these new conditions may establish novel paradigms. A promising system to do so would contain a metal with a strongly decoupled reduction potential from Ag^+^.

The Ag_2_O–MoO_3_ phase diagram contains numerous viable candidates for this including spinel Ag_2_MoO_4_, layered Ag_6_Mo_10_O_33_, orthorhombic Ag_2_Mo_3_O_10_, and two Ag_2_Mo_2_O_7_ polymorphs.^34–36^ Ag_6_Mo_10_O_33_ was reported as a cathode once in 1978 (ref. 37) and more recently has appeared as a topic of study in supercapacitor application.^38^ The electrochemistry of monoclinic and triclinic polymorphs of Ag_2_Mo_2_O_7_ has recently appeared in separate studies, where the former is capable of 825 mA h g^−1^ at 100 mA g^−1^ rates in lithium ion batteries,^39^ and the latter is capable of 190 mA h g^−1^ at 20 mA g^−1^ rates in sodium ion batteries.^40^ Collectively, these reports represent the entire body of research on the electrochemistry of silver molybdenum oxides (SMOs) at present knowledge.

Here, we find a subset of four SMO's (Ag_2_MoO_4_, monoclinic and triclinic Ag_2_Mo_2_O_7_ and Ag_2_Mo_3_O_10_·2H_2_O) which all reduce in absence of additives to form Ag^0^ early in discharge (0.05 electrons) yielding electrodes with lowered resistance by ∼100× and within an order of magnitude of each other. Even with this property, the electrodes show different capabilities to deliver voltage, capacity, and energy as a function of rate; hence, resistance is not a descriptor of materials which would thrive under low-carbon conditions. We solve for the underlying factors behind these differences and propose a descriptor to optimize Ag reductive displacement in other materials. Further, we show that when evaluated in electrodes with carbon and binder additives all SMOs achieved similar levels of performance, reinforcing the notion that composite electrodes prevent the prediction of structures which would fail in low-carbon electrodes. Our results contribute to understanding where and how Ag^+^ should be predistributed in electrodes targeting this mechanism in low-carbon applications and support future testing of carbon-free electrodes as part of battery material evaluation.

## Experimental

### Syntheses

All chemicals were reagent grade, purchased commercially, and used/stored at ambient conditions without further purification, except for CH_3_NH_3_Cl which was stored in an N_2_ glovebox. The water used in all syntheses, electrolytes and solutions was ultrapure 18 MΩ resistivity.

### β-Ag_2_MoO_4_ (1Mo)

AgNO_3_ (0.680 g, 4 mmol of Aldrich Lot MKCG5845) dissolved in 15 mL of water was added dropwise to a solution of Na_2_MoO_4_·2H_2_O (0.484 g, 2.4 mmol of Strem Lot 26713700) dissolved in 15 mL of water. The slight excess of MoO_4_^2−^ was utilized to ensure the system maintained an alkaline pH, under which Ag_2_MoO_4_ is the preferred product. On initial mixture, fine particulates are formed within a cloudy suspension with appearance of milk. Applying ∼50 °C heat for 15 minutes, the milk color disappears, and the suspension consists of filterable particulates in clear solution. The phase pure product can be isolated any time after this point. In this study, we performed an overnight reflux, collecting by filtration; 0.50–0.55 g, ∼73%. The material can be dried at 100 °C safely; we dried for 1 h.

### Monoclinic Ag_2_Mo_2_O_7_ (m-2Mo)

Reproduced from Saito *et al.*^41^ AgNO_3_ (0.488 g, 2.9 mmol) dissolved in 20 mL of water was added to 0.226 g (1.6 mmol) of MoO_3_ suspended in 20 mL of water. The suspension was refluxed for 5 h, and the resulting solid collected by filtration and washed with water. The material can be dried at 100 °C safely; we dried for 30 minutes. We were unsuccessful at preparing this polymorph by refluxing AgNO_3_ with ammonium heptamolybdate or acidified MoO_4_^2^ due to the presence of triclinic Ag_2_Mo_2_O_7_ impurity, which appears in reactions with prolonged temperatures or times; our results agree with other reports.^42,43^

### Triclinic Ag_2_Mo_2_O_7_ (t-2Mo)

Monoclinic Ag_2_Mo_2_O_7_ (200 mg) was heated to 450 °C for 5 h in air in a steel crucible and cooled to room temperature ambiently in 98% yield. The conversion mechanism appears to involve sample melting and subsequent interaction with the container before recrystallizing, as the use of new porcelain crucibles yielded samples with evenly dispersed, 4% Al dopant impurity (which were not studied further). The use of steel eliminates the impurity yet imparts a 2% loss of sample as a tarnish.

### Ag_2_Mo_3_O_10_·2H_2_O *via* (CH_3_NH_3_)_2_Mo_7_O_22_

We were not successful at isolating this material by refluxing AgNO_3_ with Mo^6+^ below pH 4, due to the persistence of kinetically favorable m-2Mo. We slightly modified the procedure of Dessapt *et al.*^44^ to better avoid the appearance of an adventitious hexagonal MoO_3_ impurity (see ESI† for details and discussion). Solutions of 1.01 g (15 mmol) of CH_3_NH_3_Cl in 5 mL of water and 1.21 g of Na_2_MoO_4_ (6 mmol) in 5 mL of water were mixed and the resultant solution was syringe filtered. 4 M HCl was added dropwise until pH 1.5 was obtained in close correlation with the appearance of a sustained white suspension. The suspension was heated in a Teflon Parr reactor, 120 °C, 6 h. The product white powder (CH_3_NH_3_)_2_Mo_7_O_22_ obtained in yield quantitative in Mo was washed with water and acetone. The reader is referred to ESI† for characterization of (CH_3_NH_3_)_2_Mo_7_O_22_ and related discussion. Subsequently, AgNO_3_ (0.235 g) was dissolved in 25 mL of 0.1 M HNO_3_, and (CH_3_NH_3_)_2_Mo_7_O_22_ (0.300 g) were added. The suspension was refluxed overnight (∼16 h) with stirring, and the yellow product was washed with water and ethanol and dried in air.

### Electrochemistry materials and methods

All tests were recorded on a Biologic VSP potentiostat. Two electrode cells were constructed inside MTI split cells, with Zn foil anode, 2 M ZnSO_4_ electrolyte, and Whatman glass fiber filter paper separator. For carbon and binder free working electrodes, silver molybdate powders were pressed between two steel foil sheets at 20 MPa into pre-massed steel gauze (Type 304, 0.001 in wire). Where applicable and mentioned in the text, composite electrodes were prepared using a slurry of silver molybdate : carbon black : PVDF binder prepared in NMP solvent with composition 8 : 1 : 1 by mass and doctor bladed onto steel foil current collector. ½′′ circles were punched from this coating and used as electrodes.

Data for galvanostatic intermittent titration technique (GITT) plots were obtained following 360 second pulses at 1e^−^/2 h rates for each material. To maintain a well-defined surface area, working electrodes in these tests were powders pressed evenly onto precut squares of graphene paper. Ionic diffusion coefficients were calculated by:^45^
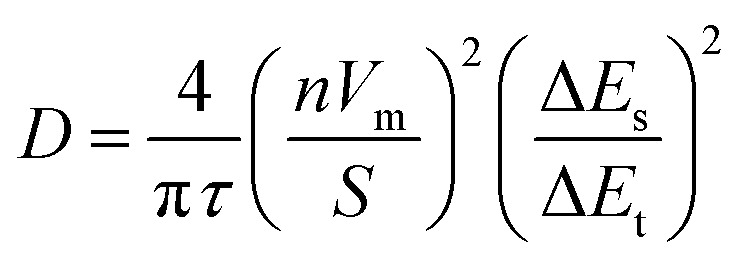
Here, *τ* = pulse length in seconds, *n* is the moles of material per electrode, *V*_m_ is the molar volume for each polymorph (59.7 cm^3^ mol^−1^1Mo, 89.6 cm^3^ mol^−1^m-2Mo, 88.4 cm^3^ mol^−1^t-2Mo and 147 cm^3^ mol^−1^3Mo), *S* is the electrode surface area (in cm^2^), Δ*E*_s_ is the difference in equilibrated voltage before and after the pulse, and Δ*E*_t_ is the difference in voltage between the end of the pulse and the equilibrated potential.

### Other materials and methods

**Fig. 1:**
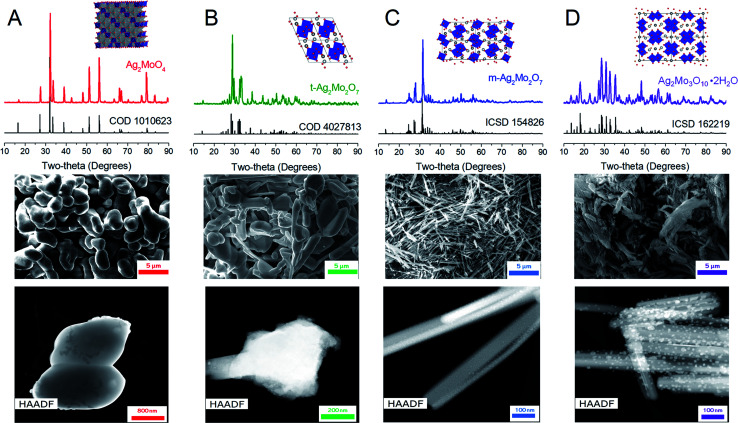
PXRD, SEM and TEM data for the materials in this work. Experimental powder diffraction patterns (colored traces) are labelled as (A) 1Mo, (B) t-2Mo, (C) m-2Mo and (D) 3Mo over structural references (black traces). In the crystal structure representations blue polyhedra represent Mo and where applicable Ag^+^ is represented by two shades of silver, differentiated by symmetry. SEM microscopy images were taken at 5000× magnification.

PXRD data were recorded on a Rigaku Miniflex with Cu source. SEM data for Fig. 1 were recorded on a JEOL JSM 6610LV, whereas data for reduced electrodes were taken with a S-Hitachi-4700-II SEM. Raman data were recorded on a Thermofisher DXR3xi Raman microscope with 532 nm laser excitation. TEM data were recorded on an FEI Talos TEM with an FEI Tecnai F20ST S/TEM. For characterization of reduced electrodes, samples were washed with 0.1 M acetic acid and water following battery disassembly. The freestanding gauze – and if applicable a steel foil support – was inserted directly into SEM and XRD for respective measurements, whereas the sample was scratched from the mesh onto a lacey carbon grid for TEM analysis.

## Results

### Syntheses and characterization

For this study disilver molybdate (Ag_2_MoO_4_, 1Mo) both known polymorphs of disilver dimolybdate (Ag_2_Mo_2_O_7_, 2Mo) and disilver trimolybdate dihydrate (Ag_2_Mo_3_O_10_·2H_2_O, 3Mo) were prepared. Literature syntheses were adapted for 1Mo, 3Mo and monoclinic m-2Mo (see Experimental). For triclinic t-2Mo, m-2Mo was heated for 5 hours at 450 °C in air; this synthesis improves upon prior reports which have prepared this polymorph *via* solid-state sintering of Ag_2_MoO_4_ or Ag_2_O with MoO_3_ requiring 2–4 days.^40,46^

Fig. 1 depicts experimental X-ray diffraction patterns demonstrating phase purity and microscopy characterization of sample morphology. Two of the samples have nanorod morphology (m-2Mo and 3Mo), and the latter exhibits a greater degree of bundling. From the Scherrer equation, the crystallite sizes were 27 and 23 nm respectively. T-2Mo and 1Mo consist of larger particulates; the former with a diverse range from rods to plates, the latter with micron sized, dense potato shapes. These crystallites were 43 and 32 nm, respectively.

High angle annular dark field scanning transmission electron microscopy (HAADF/STEM) images of all samples revealed the existence of surface particles. These were as small as 2 nm in both 2Mo, 5–15 nm in 3Mo and, in 1Mo, the particles were growing as large as 50 nm in real time during the measurement. To our knowledge, all prior studies pursuing transmission electron microscopy characterization on SMOs have reported sample instability and reduction of Ag^+^ to Ag^0^ in the beam.^41,47–51^ EDS spectroscopy confirmed that the surface particles were pure Ag; data for the most prominent samples 1Mo and 3Mo are provided as Fig. S1.† Given that no Ag^0^ peaks exist in any XRD data, we consider these results consistent with beam damage. For the images in Fig. 1, EDS verified the compositional Ag : Mo ratios expected for each sample (Fig. S2†) and SEM/EDS mapping confirmed the elements were evenly distributed on the micron scale (Fig. S3†). One rationale for the beam damage results could be the Ag^+^–Ag^+^ distances of each SMO, when compared to the product Ag^0^ crystals containing atoms 2.89 Å apart. The 2Mo samples which exhibited very small Ag^0^ particles do not have Ag^+^–Ag^+^ distances shorter than 3.36 Å. By contrast, 3Mo contains one of the shortest Ag^+^–Ag^+^ distances of any SMO in this study (3.17 Å), and in 1Mo all Ag^+^–Ag^+^ distances are 3.27 Å. Hence, Ag^0^ appears to grow more prominently in the TEM beam when the ions do not have to migrate as far.

**Fig. 2:**
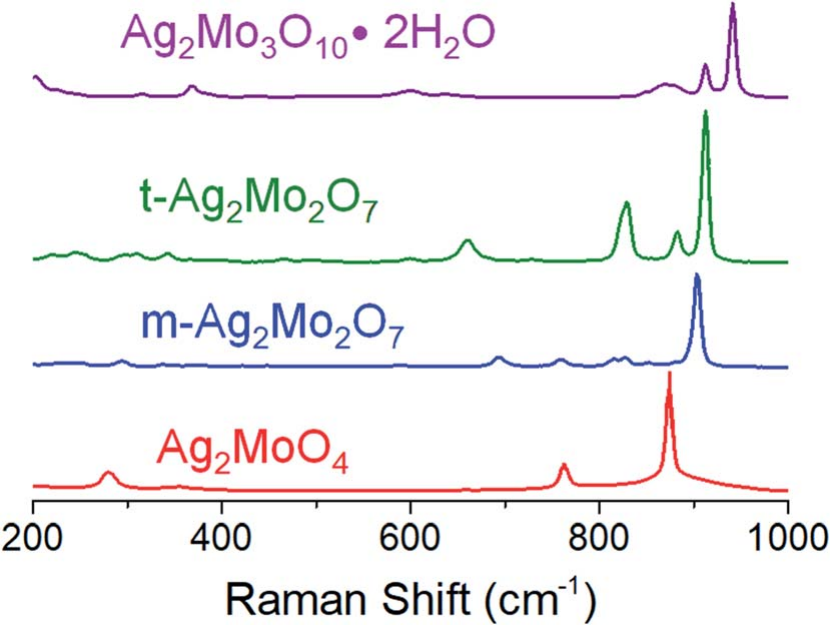
Raman spectra of the materials in this study; see text for peak values.

Fig. 2 depicts Raman spectroscopy characterization (*λ* = 532 nm). 1Mo exhibits peaks at 873, 762, 354 and 279 cm^−1^, which are in agreement within ±1 cm^−1^ to the known A_1g_, 2T_2g_ and E_g_ stretches respectively.^52,53^m-2Mo has a major peak at 905 cm^−1^ with minor bands at 853, 828, 817, 760 and 694 cm^−1^, which are in agreement within ±2 cm^−1^ to other data.^43^ We are not aware of other Raman spectroscopy data for t-2Mo, which our results find a major peak at 913 cm^−1^ with minor bands at 864, 830, 662, 344 and 311 cm^−1^. Finally, 3Mo gives a major peak at 941 cm^−1^ with minor bands at 912, 869, 636, 598, 368, 314 and 202 cm^−1^, which agrees with literature.^44^ Of particular emphasis from this dataset: larger values for the major peak at *ca.* 900 cm^−1^ indicate stronger Mo–O bonds,^54^ and by extension more acidic oxos. Our Raman data therefore confirms the expected trend MoO_4_^2−^ < Mo_2_O_7_^2−^ < Mo_3_O_10_^2−^ for increasing acidity of the molybdate frameworks.^43,55^

### Electrochemistry mechanism: reduction of Ag^+^

**Fig. 3:**
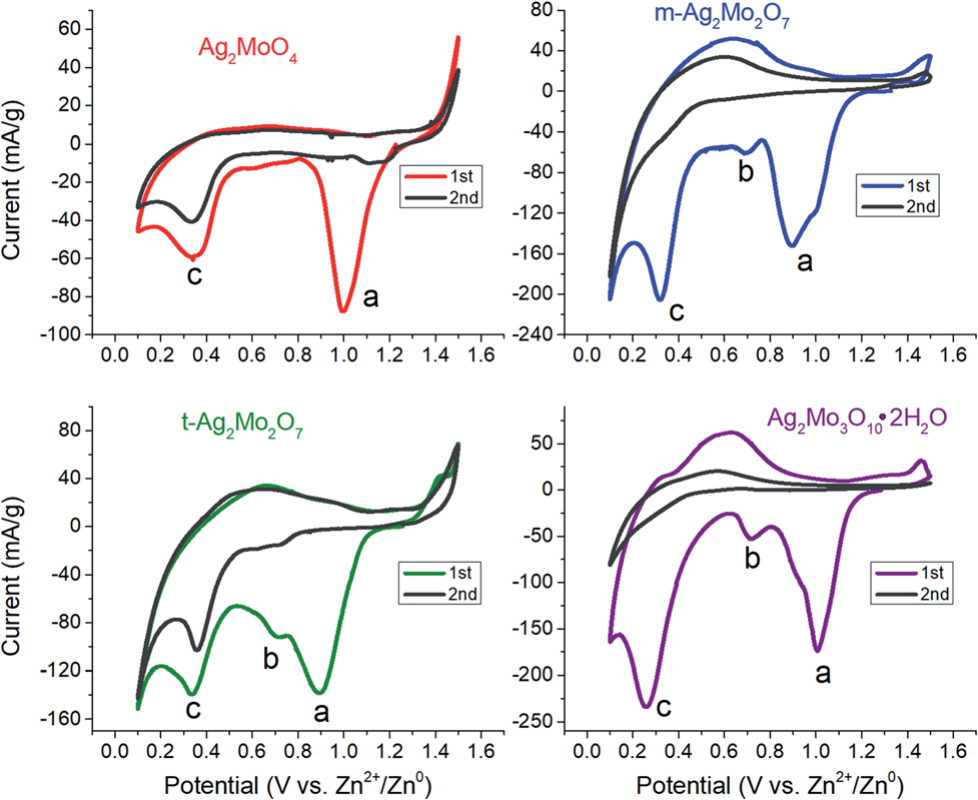
Cyclic voltammetry at 0.1 mV s^−1^ for each of the materials in this study. Conditions: 2 M ZnSO_4_ electrolyte, Zn foil anode and reference.

**Fig. 4:**
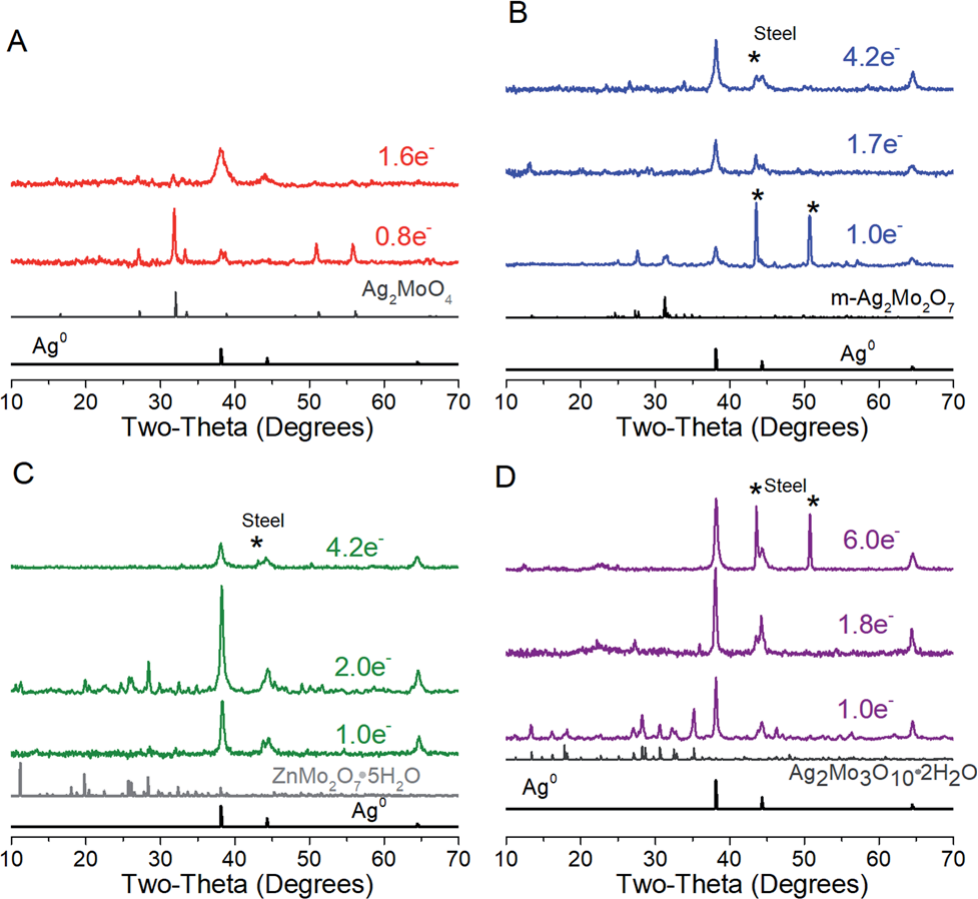
XRD characterization of electrodes reduced at 5 mA g^−1^; data are also representative up to 40 mA g^−1^. (A) Ag_2_MoO_4_, (B) m-Ag_2_Mo_2_O_7_, (C) t-Ag_2_Mo_2_O_7_, and (D) Ag_2_Mo_3_O_10_·2H_2_O.

Cyclic voltammograms of SMO powders as cathodes in 2-electrode AZIBs are provided as Fig. 3. All SMO's exhibit a cathodic peak ‘a’ located at 0.90 (2Mo) through 1.00 V (1Mo, 3Mo), which is assigned to the reduction of Ag^+^ based on several lines of evidence. (1) It is the most plausible thermodynamic match based on reduction potentials measured of Ag^+^ and Mo^6+^ standards (Fig. S4†), which occurred across 1.0–1.2 V and 0.20–0.38 V ranges *vs.* Zn^2+^/Zn^0^ respectively. (2) In all SMO's no more than 2 electron equivalents (ee) of charge per mole can be passed at peak ‘a’; this precisely matches the amount of charge needed to reduce both formula silvers quantitatively. (3) XRD (Fig. 4) identifies Ag^0^ in samples reduced by 0.8–1.0ee at the peak ‘a’ potential. (4) The Scherrer equation, when applied to the main peak at 38° 2*θ*, calculates the Ag^0^ crystallite size as increasing up to 2ee, beyond which there is no significant difference (Fig. S5†). This is consistent with the Ag^0^ network growing in this region to form crystals that ultimately exhibited a correlation of increasing size with decreasing [Ag^+^]_0_, with 6 nm in 1Mo, 17 nm for m-2Mo, 18 nm for t-2Mo and 28 nm for 3Mo. (5) Impedance data (discussed later) shows all SMO electrodes become ∼100× more conductive within 0.05ee, consistent with an expected result from forming Ag^0^*in situ*.

The peak current in CV's is known to be the combination of surface capacitive effects – which are proportional to scan rate *ν*- and bulk diffusion effects – which are proportional to *ν*^1/2^. Hence, for the overall dependence of current on scan rate *via* the equation:^56^

1 *i* = *aν*^*b*^

A plot of log(*i*) *vs.* log(*ν*) should yield a line with slope *b* between 0.5 (pure diffusion) and 1 (pure capacitance). Applied to peak ‘a’, the *b* values measured for the 2Mo and 1Mo samples (0.55–0.67, Fig. S6†) were all internally consistent with evidence of Ag^+^ reduction limited by diffusion in the bulk. However, the *b* value for 3Mo (0.88) instead suggests a more surface-limited, pseudocapacitive mechanism. In support of these electrochemistry assignments, XRD identified ZnMo_2_O_7_·5H_2_O (ICSD 245617) as the other crystalline product after 2ee reduction of t-2Mo (Fig. 4C), thereby proving a full bulk reductive displacement mechanism occurring *via*:

2 Ag_2_Mo_2_O_7_ + Zn^2+^ + 2e^−^ + 5H_2_O → 2Ag + ZnMo_2_O_7_·5H_2_O

**Fig. 5:**
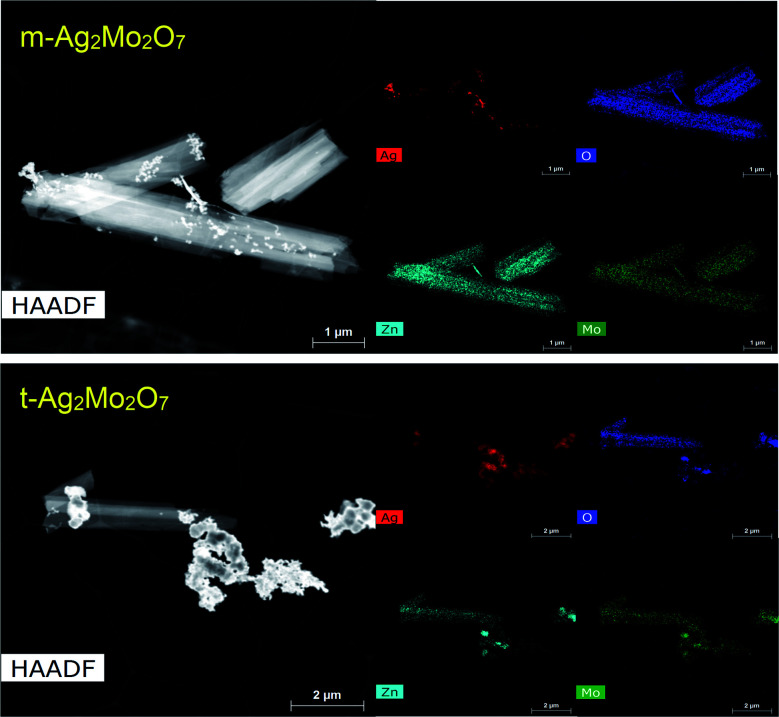
HAADF/STEM and EDS maps for m-2Mo (top) and t-2Mo (bottom) samples reduced by 2ee at 1e^−^/2 h, showing Zn displacing Ag from the initial structures.

The ZnMo_2_O_7_·5H_2_O is less crystalline than the starting t-2Mo (25 *vs.* 43 nm, respectively). Attempts to detect a ZnMo_2_O_7_ phase from reduction of less-crystalline m-2Mo were unsuccessful but HAADF/STEM characterization (Fig. 5) provides evidence further confirming a closely related mechanism. In these images both 2ee reduced 2Mo samples were found to contain particles analyzing as pure Ag in addition to sheets clearly characterized by EDS as the zinc-molybdenum oxide (Fig. 5, S7 and S8†). SEM-EDS mapping on the micron scale (Fig. S9†), further confirms the primarily bulk mechanism for m-2Mo.

**Fig. 6:**
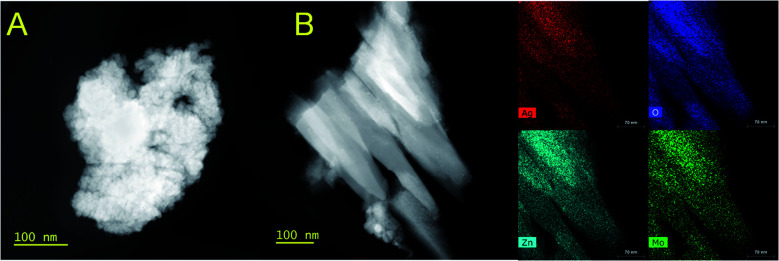
HAADF/STEM and EDS maps for 1Mo reduced by 1.6ee at 1e^−^/2 h. (A) The morphology of a pure Ag aggregate. (B) Sheets of ZnMoO_*x*_ with residual silver existing as ultrafine particles; the yellow box was analyzed by EDS.

Reduced 1Mo electrodes consisted of two types of particles: aggregates of pure nanosized Ag (Fig. 6A), and larger sheets with Ag : Zn : Mo of 1 : 2 : 2 (Fig. 6B). The sheets were covered in ultrafine, ∼2 nm particles resembling the Ag in the aggregates (Fig. S10†), though EDS mapping does not assign the total Ag concentration detected to solely those particles. The close spatial association of Zn with Mo, combined with clear evidence of Ag separate from Mo seen also by SEM (Fig. S11†), is also consistent with a bulk reduction displacement mechanism of:

3 Ag_2_MoO_4_ + Zn^2+^ + 2e^−^ → 2Ag + ZnMoO_4_

**Fig. 7:**
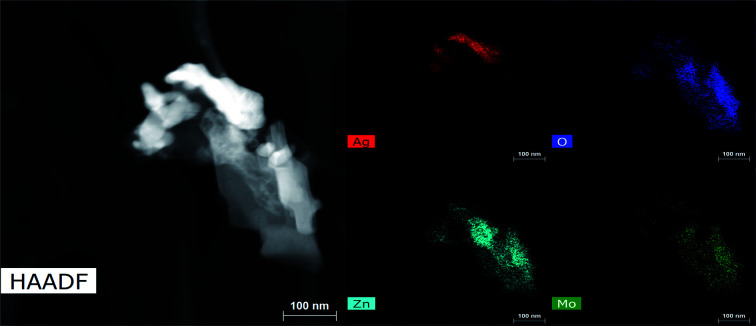
HAADF/STEM and EDS maps for 3Mo reduced by 2ee at 1e^−^/2 h, showing regions of Zn + O mixed with Ag. The Zn : Mo ratio is 2.3 : 1.

We repeatedly detected Ag^0^ as the only crystalline product from reduction of 3Mo. Microscopy images of reduced 3Mo electrodes are shown in Fig. 7 and S12† and were consistent with the absence of a bulk mechanism by electrochemistry. In this data, the major samples detected were sheets with high concentrations of Zn + O, with Mo existing in low concentrations and in distributions not matching Zn. The Zn + O sheets were either in direct contact or closely associated with large domains of Ag, which confirmed the XRD data detecting increased Ag^0^ crystallinity. These results overall do not provide evidence for substitution of Zn into the Mo_3_O_10_^2−^ framework. Rather, the data suggests the existence of a silver-coated basic zinc salt (*e.g.*, Zn_4_SO_4_(OH)_6_), which is a known product from Zn/MoO_3_ and numerous other Zn electrochemistries^57^ as resulting from a mechanism in which the pH of the electrolyte increases. We had some success detecting a pH increase in ZnCl_2_ electrolyte, which allowed the identification of simonkoellite Zn_5_(OH)_8_Cl_2_ H_2_O on the anode (Fig. S13†) by PXRD.

The close association of Ag^0^ with the basic zinc salt observed both by TEM and SEM (Fig. S14†) suggests a 3Mo reduction mechanism which increases the electrolyte's pH locally near where Ag^0^ forms. Here, we propose:

4 Ag_2_Mo_3_O_10_·2H_2_O + 2e^−^ → 2Ag + 3MoO_3_ + H_2_O + 2OH^−^

5 Zn^2+^ + *x*SO_4_^2−^ + (2 − 2*x*)OH^−^ → Zn(SO_4_)_*x*_(OH)_2−2*x*_

### Electrochemistry mechanism, subsequent steps

We observed two reduction steps: a minor one at 0.7 V and a major one at ∼0.3 V (labeled as (b) and (c) respectively) in CV's of both 2Mo and 3Mo following the consumption of their Ag^+^ ions (Fig. 3).

Given that aqueous Mo^6+^ standards reduce at 0.3 V (Fig. S4†), and that peak ‘b’ cannot account for quantitative reduction of Mo^6+^ in any sample, peak ‘c’ is assigned to the reduction of Mo^6+^.

The assignment of peak ‘b’ occurring for a fraction of the Mo^6+^ is developed by considering that it is most prominent in t-2Mo and absent from the CV of 1Mo. The only redox-active component in the t-2Mo electrode capable of accounting for peak ‘b’ is ZnMo_2_O_7_·5H_2_O, which is a layered structure of Mo_2_O_7_^2−^ chains linked by Zn^2+^ cations.^58^ Other layered MoO_3_ AZIB cathodes are well-known to exhibit peaks at 0.7 V and ∼0.3 V.^57,59^ By contrast, the proposed stoichiometry of the phase in the 1Mo sample is ZnMoO_4_, for which all known crystalline polymorphs^60,61^ are not layered. Therefore, peak ‘b’ is proposed to indicate the existence of layered domains in the Mo^6+^ oxide produced following reduction of each sample's silver ions.

Prior literature has shown the duration of each potential step at 0.7 V and 0.3 V for layered Mo^6+^ cathodes is ascribed to the increasing effects of H_3_O^+^ rather than Zn^2+^ as the charge carrier.^57,59^ We utilized the galvanostatic intermittent titration technique (GITT) to characterize peaks ‘b’–‘c’ since Zn^2+^ diffusion into MoO_3_ (10^−10^ to 10^−12^ cm^2^ s^−1^)^59,62^ is known to be slower than H^+^ (10^−8^ to 10^−9^ cm^2^ s^−1^).^59^ For all SMO's, we measured ion diffusion coefficients within the range of 10^−11^ to 10^−12^ cm^2^ s^−1^ while on the Ag^+^ reduction plateau, which is where either Zn diffusion or growth is confirmed by microscopy (Fig. S15†). In direct correlation with the drop to lower potentials for peaks ‘b’–‘c’, the ionic diffusion coefficients increased by *ca.* 2 orders of magnitude. Therefore, Zn^2+^ clearly charge balances the reduction of 2Ag^+^ and, once complete, H^+^ is proposed to charge balance the reduction of the resulting ZnMoO_*x*_ or MoO_*x*_.

Under our conditions the SMO's exhibited poor cyclability by CV (Fig. 3) and most other methods tested by our group. Both 2Mo and 3Mo show an anodic curve at 0.6 V, assigned to the reoxidation of a fraction of the reduced Mo, which is also observed for aqueous Mo^6+^ standards. In all SMO's the anodic peak for Ag^0^ initiates at 1.5 V but to prevent a significant loss of the electronic conductive network we bounded CVs at that potential. Subsequent scans showed no appreciable current for the reduction of Ag^+^. Our findings are largely in agreement with the consensus that MoO_3_ in aqueous batteries exhibits poor capacity retention upon cycling,^63^ with evidence so far suggesting that this is due to the dissolution of reduced Mo which is known from aqueous electrochemistry of Mo oxide passive layers.^64^ Numerous reports have indicated generally underwhelming metrics for aqueous MoO_3_ battery or capacitor electrochemistry unless polypyrrole composites, gel electrolytes, or water-in-salt electrolytes are utilized to stop dissolution;^57,63,65–69^ these strategies lie outside the scope of this study.

### Metrics for Ag^+^ reduction

**Fig. 8:**
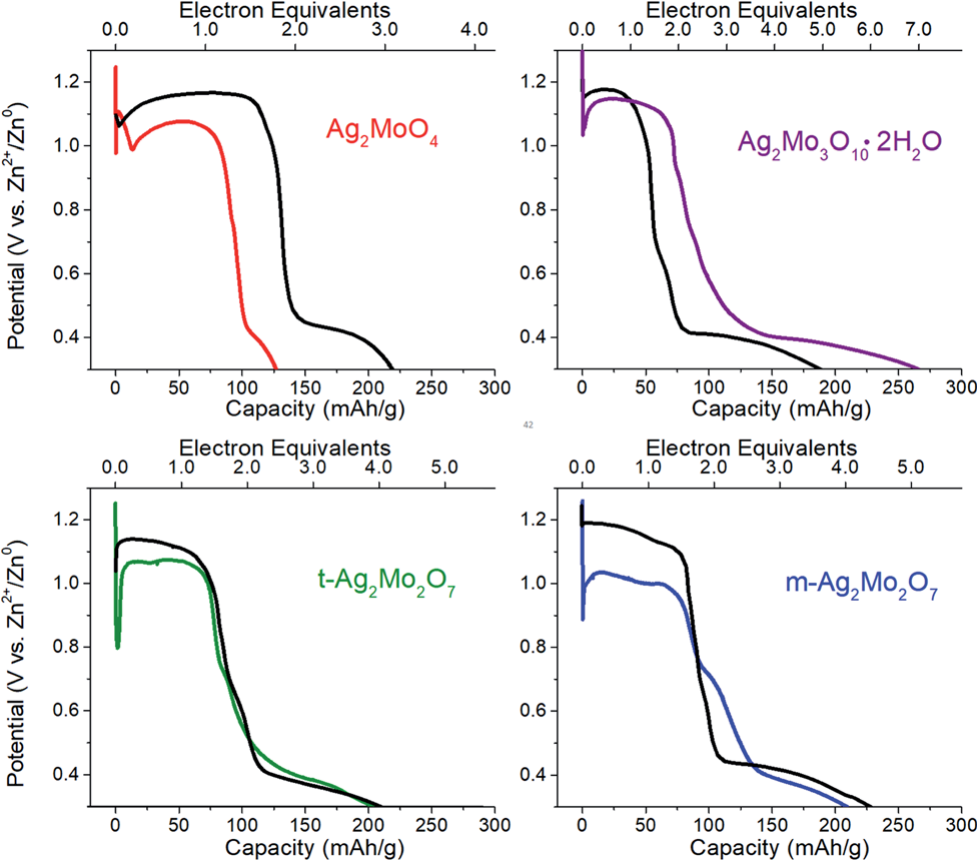
Representative galvanostatic discharge at 40 mA g^−1^ rates for the materials in this study. Working electrodes are the pure powder pressed into steel mesh (colored traces: red 1Mo, green t-2Mo, blue m-2Mo and purple 3Mo) or a composite electrode with 80/10/10 active material/carbon/PVDF composition (black traces). Conditions: 2 M ZnSO_4_ electrolyte, Zn foil anode and reference.

We compared the electrochemical performance of SMO's as stand-alone pellets (Fig. 8, colored traces) *vs.* composite electrodes containing carbon predistributed (Fig. 8, black traces) at 40 mA g^−1^ rates. Notably, the stand-alone pellets all exhibit an initial voltage drop followed by a recovery – a property well known in SVO cathodes and analogs^31,70,71^ as the initial polarization being overcome by the *in situ* generation of a conductivity change provided by the Ag^0^ network. We consider the following metrics:

#### (1) Operating voltage plateau

With a carbon network able to lower interparticle resistance, the initial voltage drop/recovery is mitigated in all cases. The resulting reduction of silver is sustained at 1.17 ± 0.02 V, crucially irrespective of the SMO structure. However, without carbon only 3Mo is capable of attaining >1.1 V for the duration of the plateau; by contrast, 1Mo and both 2Mo are least 50 mV lower.

#### (2) Capacity at high voltage

For all SMO's with carbon the 2e^−^ reduction level occurs as the voltage falls from the 1.17 V plateau. While both 2Mo and 3Mo reach this boundary without carbon, 1Mo does not discharge by 2e^−^ without carbon.

#### (3) Capacity at low voltage

With Mo^4+^ as the lowest oxidation state known in Zn/MoO_3_ batteries, plausible upper bound capacities are 4 electrons in 1Mo (284 mA h g^−1^), 6 electrons in 2Mo (312 mA h g^−1^) and 8 electrons in 3Mo (304 mA h g^−1^). Experimentally, we measured 3Mo (89%, 270 mA h g^−1^) > m-2Mo (67%, 210 mA h g^−1^) ≈ t-2Mo (65%, 200 mA h g^−1^) ≫ 1Mo (45%, 130 mA h g^−1^) when ranking the percentage delivered by carbon-free electrodes above 0.3 V. 3Mo significantly underperforms in the composite electrode suggesting the binder is inhibiting this material's more pseudocapacitive mechanism.

**Fig. 9:**
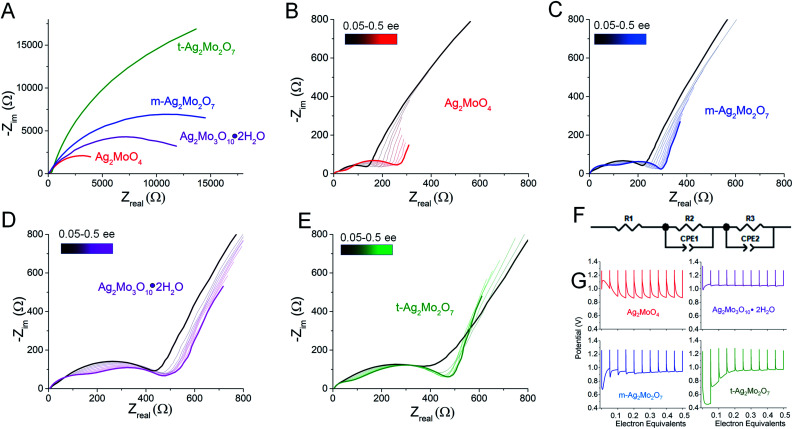
(A) Representative Nyquist plots of pellet electrodes; (B–E) Nyquist plots following titration of 0.05 electron equivalents into each SMO at 40 mA g^−1^; (F) the circuit used to fit the two semicircles in the impedance data. (G) Potential *vs.* electron equivalents at 40 mA g^−1^ rates.

For greater resolution on the formation of the Ag^0^ network in stand-alone pellets pulse tests were performed, where 10 × 0.05 electron equivalent intervals were titrated into each SMO; electrodes were relaxed to a stable OCV between pulses. Fig. 9A depicts representative Nyquist plots of SMO electrodes prior to any reduction. For quantitative fit, the semicircles were modeled *via* a Randles-type circuit with two *R*/*C* parallels, (Fig. 9F), which has been used for other silver metal oxides.^1,3,31,72^ In this circuit, *R*_1_ is the solution resistance, the *R*_2_/CPE parallel models the anode and the *R*_3_/CPE parallel models the cathode. *R*_3_ is of interest as it represents the interparticle charge transfer resistance, *R*_ct_. Results from fittings quantify the initial *R*_ct_ ranging 9–45 kΩ.

The electrode potentials were monitored during each 0.05 electron pulse (Fig. 9G). Fig. 9B–E depict that, upon relaxation after the first 0.05 electron equivalent reduction, all SMO's exhibit a compressed semicircle indicative of a decreased charge transfer resistance. *R*_ct_ is lower than 530 Ω in all cases, representing a resistance decrease of up to 100×, with the largest decrease from the t-2Mo electrode.

The first 0.05ee is sufficient to generate enough Ag^0^ to recover the voltage in 1Mo, 3Mo and m-2Mo before the end of pulse duration (Fig. 9G). That t-2Mo requires additional Ag^0^ is not ascribed to its morphology but rather its initially highest *R*_ct_, which causes a higher polarization to need to overcome. The data in Fig. 9G clearly show that the initial voltage drop of the electrode is proportional to the initial resistance; we observed similar trends in galvanostatic reduction (Fig. S16†).

In comparison to the 0–0.05ee decrease, subsequent titration of 0.05–0.5ee changed *R*_ct_ far less significantly. In particulate samples *R*_ct_ gets slightly worse: 1Mo increased from 141 to 275 Ω (Fig. 9B), correlating with an electrode which operated at 0.87–0.90 V in each pulse. For t-2Mo (Fig. 9E) the increase observed was 390–465 Ω, but this primarily occurred in early electron increments and ultimately correlated with higher pulse voltages of 0.97 V. m-2Mo mostly retained the same *R*_ct_ (230–220 Ω, Fig. 9C) and exhibited a comparable 0.94 V pulse voltage (Fig. 9G). Finally, 3Mo recovered voltage to the highest plateau at 1.05 V, but this correlated with a relatively high *R*_ct_ that was consistently improving with each pulse from 528 to 385 Ω (Fig. 9D).

Collectively our results show there is no association of low *R*_ct_ with operational pulse voltage, which varies for Ag^+^ reduction by as much as 140 mV. The clear potential order 1Mo < 2Mo < 3Mo from pulse tests matches that from galvanostatic reduction (point 1). Given that each SMO appears to form a sufficient Ag^0^ network to fully recover voltage within 0.2ee, we evaluated the silver networks by two more metrics:

#### (4) Rate capability

**Fig. 10:**
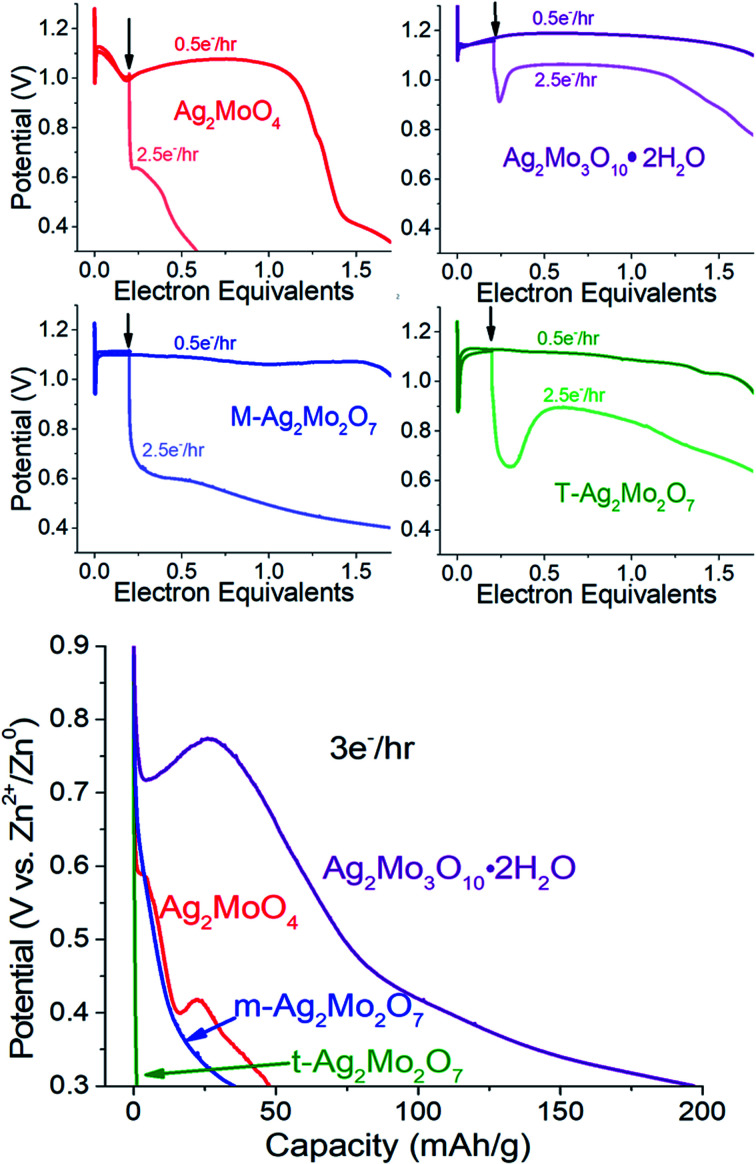
Top: The SMOs are discharged at 0.5e^−^ h^−1^ rates until the position of the arrow at 0.2ee. Light curves indicate a rate change to 2.5e^−^ h^−1^ while the dark curve indicates the remainder of discharge at 0.5e^−^h^−1^. Bottom: Representative galvanostatic discharge at 3e^−^ h^−1^ for the materials in this study.

We reduced each carbon-free SMO by 0.2 electrons at 0.5e^−^ h^−1^ rates to form a Ag^0^ network. Then, the reduction rate was increased by a factor of 5. Over the next 1.5 electrons – the upper bound for comparison – the resulting electrodes all deliver a fraction of the W h kg^−1^ energy relative to when the rate is unmodified. For the sample data shown in Fig. 10, top, this fraction was 84% (3Mo) > 73% (t-2Mo) > 48% (m-2Mo) > 15% (1Mo).

#### (5) Formation rate kinetics of Ag^0^

We initiated reduction of each carbon-free SMO at 3e^−^ h^−1^; a 6× faster rate than the prior test (Fig. 10, bottom). T-2Mo, which needs more Ag^0^ to overcome an intrinisically higher *R*_ct_, fails this test most prominently. At the other extreme, 3Mo delivered 200 mA h g^−1^, which is 86% of the value from the 6× slower rate.

## Discussion

In the design of cathodes capable of displacing Zn^2+^ for 2Ag^+^ in aqueous conditions, the above data (points 1–5) suggests a trend of 1Mo ≪ m-2Mo ≤ t-2Mo for the most optimized initial structure and the “best” Ag^0^ network at replicating carbon composites. However, much superior metrics are obtained by 3Mo, which does not perform Zn^2+^/2Ag^+^ bulk substitution directly nor yields electrodes with lowest *R*_ct_. Such a trend is not addressed by morphology nor crystallinity because both are comparable to the worse-performing m-2Mo. Rather, the trend appears easily explained in the context of the acidity of the molybdate framework by Raman spectroscopy (Fig. 2). The consequence of a molybdate framework with greater acidity is that the Ag^+^ in 3Mo have longer Ag–O bonds 2.44–2.88 Å relative to m-2Mo (2.33–2.64 Å), t-2Mo (2.27–2.76 Å), and especially 1Mo (2.31 Å). With the Ag^+^ ions most weakly bound to oxygen, the energy penalty for Ag–O bond breakage is lowered and results in improved access to Ag^+^ with minimal voltage loss and fast kinetics.

The tradeoff that naturally follows by making a cation easier to displace from a framework is that it becomes harder to put a new cation in. As our measurements show, this tradeoff in molybdates is at first surmountable (because 2Mo > 1Mo) but then is avoided entirely through a charge balancing mechanism switch (3Mo). Hence, our results appear to demonstrate the existence of a “crossover” acidity below which Ag^+^ can be made more accessible and above which Zn^2+^ insertion no longer occurs. In AZIBs this crossover thus occurs between the acidities of Mo_2_O_7_^2−^ and Mo_3_O_10_^2−^ frameworks. As molecular species, it is intriguing that 10 mM solutions give pH's of 4.58 and 3.52 respectively^55^ which appear to straddle the typical ∼4.0–4.5 pH of aqueous ZnSO_4_.

Prior reports on nonaqueous Li/SVO cells do not appear to mention this concept, though there are striking comparisons. Several reports qualitatively correlated materials with higher Ag^+^ content promoting overall higher voltage but with a tradeoff of lower capacity.^24,73,74^ This correlation doesn't describe structures, but our Zn/SMO composite electrode results agree with the conceptual trend. Chen *et al.* proposed silver ions in SVO interlayers were more facile to reduce than silver ions as structured octahedra.^75^ In our study the silver ions in both 2Mo and 3Mo qualify as “interchain”, compared to the structurally rigid Ag^+^ in 1Mo, but with an observed order of 1Mo < 2Mo < 3Mo, we note this description appears conceptually correct but incomplete. Therefore, our results not only appear consistent with prior trends in nonaqueous electrochemistry but also provide an overall revision.

Our data do not rule out the solvation of Ag^+^ with water molecules unique to 3Mo as another possible descriptor causing or aiding a change in mechanism. However, a kinetic advantage to weakly-bound Ag^+^ sites has been noted in nonaqueous chemistry recently: purposeful placement of Ag^+^ on the surface of Ag_*x*_Mn_8_O_16_ nanowires improves fast-rate discharge capacity by 2-fold compared to when the Ag^+^ was added to the bulk MnO_*x*_ tunnel structure.^28,72^ We propose, therefore, that this design principle has applications beyond aqueous electrolyte and Zn^2+^ ions.

## Conclusions

This investigation was motivated by needs to understand reduction displacement mechanisms under aqueous conditions and to establish the impacts of varying the initial distribution, concentration, and structure of Ag^+^ in mixed metal oxides. We have found across a systematic electrochemical study of four silver molybdates that less than 0.2 electron equivalents (10% of Ag^+^) need be reduced to reach locally low values of *R*_ct_ in all cases, and while these values are ultimately comparable in magnitude irrespective of initial structure, they are not predictive of materials which are robust to rate changes nor those which can minimize losses when reducing the carbon content of electrodes. The best descriptor for this is the increase of acidity of molybdate frameworks, which first causes improved displacement of Ag^+^ for Zn^2+^ in aqueous electrolyte before forcing an alternative pseudocapacitive mechanism of charge balance to predominate. This factor contributed more than differences in crystallinity or morphology across the samples.

All these materials and mechanisms deliver comparable voltage for the Ag^+^ reduction when utilized in composite electrodes containing carbon and binder, but at least one case exists (3Mo) where additives are in fact detrimental to the mechanism. So, while composite electrodes are necessary to advance the electrochemistry of new Zn^2+^ hosts and attain good individual metrics, we have shown here that they potentially can improve the performance of materials with fundamental weaknesses that only research in efforts to increase functional percentages in the next generation of electrochemical energy storage devices will expose.

## Conflicts of interest

There are no conflicts to declare.

## Supplementary Material

RA-011-D1RA07765A-s001

## References

[cit1] Bock D. C., Bruck A. M., Pelliccione C. J., Zhang Y., Takeuchi K. J., Marschilok A. C., Takeuchi E. S. (2016). RSC Adv..

[cit2] Knehr K. W., West A. C. (2016). J. Electrochem. Soc..

[cit3] Kirshenbaum K. C., Bock D. C., Zhong Z., Marschilok A. C., Takeuchi K. J., Takeuchi E. S. (2014). Phys. Chem. Chem. Phys..

[cit4] Kirshenbaum K., Bock D. C., Lee C.-Y. C.-Y., Zhong Z., Takeuchi K. J., Marschilok A. C., Takeuchi E. S. (2015). Science.

[cit5] Kirshenbaum K. C., Bock D. C., Brady A. B., Marschilok A. C., Takeuchi K. J., Takeuchi E. S. (2015). Phys. Chem. Chem. Phys..

[cit6] Bock D. C., Marschilok A. C., Takeuchi K. J., Takeuchi E. S. (2012). Electrochim. Acta.

[cit7] Kundu D., Adams B. D., Duffort V., Vajargah S. H., Nazar L. F. (2016). Nat. Energy.

[cit8] Konarov A., Voronina N., Jo J. H., Bakenov Z., Sun Y.-K., Myung S.-T. (2018). ACS Energy Lett..

[cit9] Li H., Ma L., Han C., Wang Z., Liu Z., Tang Z., Zhi C. (2019). Nano Energy.

[cit10] Lan B., Peng Z., Chen L., Tang C., Dong S., Chen C., Zhou M., Chen C., An Q., Luo P. (2019). J. Alloys Compd..

[cit11] Shan L., Yang Y., Zhang W., Chen H., Fang G., Zhou J., Liang S. (2019). Energy Storage Mater..

[cit12] Guo S., Fang G., Liang S., Chen M., Wu X., Zhou J. (2019). Acta Mater..

[cit13] Li Q., Liu Y., Ma K., Yang G., Wang C. (2019). Small Methods.

[cit14] Zeng J., Chao K., Wang W., Wei X., Liu C., Peng H., Zhang Z., Guo X., Li G. (2019). Inorg. Chem. Front..

[cit15] Liu H., Wang J., Sun H., Li Y., Yang J., Wei C., Kang F. (2020). J. Colloid Interface Sci..

[cit16] Liu Y., Li Q., Ma K., Yang G., Wang C. (2019). ACS Nano.

[cit17] Yu X., Hu F., Cui F., Zhao J., Guan C., Zhu K. (2020). Dalton Trans..

[cit18] Yu X., Hu F., Guo Z.-Q. Q., Liu L., Song G.-H. H., Zhu K. (2021). Rare Met..

[cit19] Yang Y., Tang Y., Liang S., Wu Z., Fang G., Cao X., Wang C., Lin T., Pan A., Zhou J. (2019). Nano Energy.

[cit20] Chae M. S., Attias R., Dlugatch B., Gofer Y., Aurbach D. (2021). ACS Appl. Energy Mater..

[cit21] Shan L., Zhou J., Han M., Fang G., Cao X., Wu X., Liang S. (2019). J. Mater. Chem. A.

[cit22] Chen L., Yang Z., Wu J., Chen H., Meng J. (2020). Electrochim. Acta.

[cit23] Zhang Y., Chen A., Sun J. (2021). J. Energy Chem..

[cit24] Takeuchi K. J., Marschilok A. C., Davis S. M., Leising R. A., Takeuchi E. S. (2001). Coord. Chem. Rev..

[cit25] Belardi G., Ballirano P., Ferrini M., Lavecchia R., Medici F., Piga L., Scoppettuolo A. (2011). Thermochim. Acta.

[cit26] Accardo A., Dotelli G., Musa M. L., Spessa E. (2021). Appl. Sci..

[cit27] Li L., Fan E., Guan Y., Zhang X., Xue Q., Wei L., Wu F., Chen R. (2017). ACS Sustainable Chem. Eng..

[cit28] Smith P. F., Brady A. B., Lee S. Y., Bruck A. M., Dooryhee E., Wu L., Zhu Y., Takeuchi K. J., Takeuchi E. S., Marschilok A. C. (2018). ACS Appl. Mater. Interfaces.

[cit29] Huang J., Hu X., Brady A. B., Wu L., Zhu Y., Takeuchi E. S., Marschilok A. C., Takeuchi K. J. (2018). Chem. Mater..

[cit30] Kirshenbaum K. C., Bock D. C., Lee C.-Y., Zhong Z., Takeuchi K. J., Marschilok A. C., Takeuchi E. S. (2015). Science.

[cit31] Huie M. M., Bock D. C., Zhong Z., Bruck A. M., Yin J., Takeuchi E. S., Takeuchi K. J., Marschilok A. C. (2017). J. Electrochem. Soc..

[cit32] Huang J., Poyraz A. S., Lee S.-Y., Wu L., Zhu Y., Marschilok A. C., Takeuchi K. J., Takeuchi E. S. (2017). ACS Appl. Mater. Interfaces.

[cit33] Wang L., Wu Q., Abraham A., West P. J., Housel L. M., Singh G., Sadique N., Quilty C. D., Wu D., Takeuchi E. S., Marschilok A. C., Takeuchi K. J. (2019). J. Electrochem. Soc..

[cit34] Gatehouse B., Leverett P. (1969). Chem. Commun..

[cit35] Wyckoff R. W. G. (1922). J. Am. Chem. Soc..

[cit36] Lasocha W., Jansen J., Schenk H. (1994). J.
Solid State Chem..

[cit37] Bonino F., Lazzari M., Scrosati B. (1978). J. Electroanal. Chem..

[cit38] Kumar V., Matz S., Hoogestraat D., Bhavanasi V., Parida K., Al-Shamery K., Lee P. S. (2016). Adv. Mater..

[cit39] Zhang M., Gao Y., Chen N., Ge X., Chen H., Wei Y., Du F., Chen G., Wang C. (2017). Chem.–Eur. J..

[cit40] Chen N., Gao Y., Zhang M., Meng X., Wang C., Wei Y., Du F., Chen G. (2016). Chem.–Eur. J..

[cit41] Saito K., Kazama S., Matsubara K., Yui T., Yagi M. (2013). Inorg. Chem..

[cit42] Singh D. P., Sirota B., Talpatra S., Kohli P., Rebholz C., Aouadi S. M. (2012). J. Nanopart. Res..

[cit43] Hakouk K., Lajaunie L., El Bekkachi H., Humbert B., Arenal R. (2018). J. Mater. Chem. C.

[cit44] Hakouk K., Deniard P., Lajaunie L., Guillot-Deudon C., Harel S., Wang Z., Huang B., Koo H.-J., Whangbo M.-H., Jobic S., Dessapt R. (2013). Inorg. Chem..

[cit45] Weppner W., Huggins R. A. (1977). J. Electrochem. Soc..

[cit46] Wenda E. (1998). J. Therm. Anal..

[cit47] Cui X., Yu S. H., Li L., Biao L., Li H., Mo M., Liu X. M. (2004). Chem.–Eur. J..

[cit48] Feng M., Zhang M., Song J. M., Li X. G., Yu S. H. (2011). ACS Nano.

[cit49] Ng C. H. B., Fan W. Y. (2015). Cryst. Growth Des..

[cit50] Fabbro M. T., Saliby C., Rios L. R., La Porta F. A., Gracia L., Li M. S., Andrés J., Santos L. P. S., Longo E. (2015). Sci. Technol. Adv. Mater..

[cit51] Cunha F. S., Sczancoski J. C., Nogueira I. C., de Oliveira V. G., Lustosa S. M. C., Longo E., Cavalcante L. S. (2015). CrystEngComm.

[cit52] Moura J. V. B., Silva J. G., Freire P. T. C., Luz-lima C., Pinheiro G. S., Viana B. C., Filho J. M., Souza-filho A. G., Saraiva G. D. (2016). Vib. Spectrosc..

[cit53] Gouveia A. F., Sczancoski J. C., Ferrer M. M., Lima A. S., Santos M. R. M. C., Li M. S., Santos R. S., Longo E., Cavalcante L. S. (2014). Inorg. Chem..

[cit54] Hardcastle F. D., Wachs I. E. (1990). J. Raman Spectrosc..

[cit55] Nagul E. A., Mckelvie I. D., Worsfold P., Kolev S. D. (2015). Anal. Chim. Acta.

[cit56] Wang J., Polleux J., Lim J., Dunn B. (2007). J. Phys. Chem. C.

[cit57] Wang L., Yan S., Quilty C. D., Kuang J., Dunkin M. R., Ehrlich S. N., Ma L., Takeuchi K. J., Takeuchi E. S., Marschilok A. C. (2021). Adv. Mater. Interfaces.

[cit58] Grzywa M., Łasocha W., Surga W. (2007). J. Solid State Chem..

[cit59] Zhang H., Wu W., Liu Q., Yang F., Shi X., Liu X., Yu M., Lu X. (2021). Angew. Chem., Int. Ed..

[cit60] Abrahams S. C. (1967). J. Chem. Phys..

[cit61] Mtioui-Sghaier O., Mendoza-Meroño R., Ktari L., Dammak M., García-Granda S. (2015). Acta Crystallogr., Sect. E: Crystallogr. Commun..

[cit62] Xiong T., Zhang Y., Wang Y., Lee W. S. V., Xue J. (2020). J. Mater. Chem. A.

[cit63] He X., Zhang H., Zhao X., Zhang P., Chen M., Zheng Z. (2019). Adv. Sci..

[cit64] Saji V. S., Lee C. W. (2012). ChemSusChem.

[cit65] Zhang X., Zeng X., Yang M., Qi Y. (2014). ACS Appl. Mater. Interfaces.

[cit66] Liu Y., Zhang B., Yang Y., Chang Z., Wen Z., Wu Y. (2013). J. Mater. Chem. A.

[cit67] Liu Y., Zhang B. H., Xiao S. Y., Liu L. L., Wen Z. B., Wu Y. P. (2014). Electrochim. Acta.

[cit68] Tang W., Liu L., Zhu Y., Sun H., Wu Y., Zhu K. (2012). Energy Environ. Sci..

[cit69] Wang F., Liu Z., Wang X., Yuan X., Wu X., Zhu Y., Fu L., Wu Y. (2016). J. Mater. Chem. A.

[cit70] Takeuchi E. S., Marschilok A. C., Tanzil K., Kozarsky E. S., Zhu S., Takeuchi K. J. (2009). Chem. Mater..

[cit71] Marschilok A. C., Kozarsky E. S., Tanzil K., Zhu S., Takeuchi K. J., Takeuchi E. S. (2010). J. Power Sources.

[cit72] Zhang B., Smith P. F., Lee S.-Y., Wu L., Zhu Y., Takeuchi E. S., Marschilok A. C., Takeuchi K. J. (2017). J. Electrochem. Soc..

[cit73] Takeuchi E. S., Piliero P. (1987). J. Power Sources.

[cit74] Sorensen E. M., Izumi H. K., Vaughey J. T., Stern C. L., Poeppelmeier K. R. (2005). J. Am. Chem. Soc..

[cit75] Zhang S., Li W., Li C., Chen J. (2006). J. Phys. Chem. B.

