# Supplementary material for: Reduction of silver ions in molybdates: elucidation of framework acidity as the factor controlling charge balance mechanisms in aqueous zinc-ion electrolyte
Source: RSC Adv. 2021 Dec 13;11(62):39523–33. doi: 10.1039/d1ra07765a (PMC9044464; doi:10.1039/d1ra07765a)
Supplement: RA-011-D1RA07765A-s001 [file RA-011-D1RA07765A-s001.pdf]

Electronic Supplementary Material (ESI) for RSC Advances.
This journal is © The Royal Society of Chemistry 2021

Supporting information for:

Reduction of Silver Ions in Molybdates: Elucidation of Framework Acidity as the Factor Controlling Charge Balance Mechanisms in Aqueous Zinc-Ion Electrolyte

*Derrick Combs[1], Brendan Godsel[1], Julie Pohlman-Zordan[1], Allen Huff[1], Jackson King[1], Rob Richter[2], and Paul F. Smith[1]**

[1]Department of Chemistry, Valparaiso University, 1710 Chapel Drive, Valparaiso IN 46383

[2]Department of Chemistry and Physics, Chicago State University, 9501 S. King Drive, Chicago IL 60628

Material	Electrolyte	Voltage (V)	Specific Capacity (first, mAh/g)	Retention %/cycle	Ref
$Ag_{0.33}V_2O_5$	2 M $Zn(CF_3SO_3)_2$	0.2-1.6	418 (0.2 A/g)	~60%/100 @ 0.5A/g	[1]
$Ag_{0.4}V_2O_5$	3M $ZnSO_4$	0.4-1.4	~320 (0.5 A/g)	~70%/1000 @ 5A/g	[2]
$Ag_{0.33}V_2O_5$	2M $ZnSO_4$	0.4-1.4	350 (0.05 A/g)	83%/100 @ 1A/g	[3]
$Ag_{1.2}V_3O_8$	2M $ZnSO_4$	0.4-1.4	350 (0.05 A/g)	~80%/100 @1A/g	[3]
$Ag_2V_4O_{11}$	2M $ZnSO_4$	0.4-1.4	~240 (0.1 A/g)	~66%/100 @ 1A/g	[3]
β-$AgVO_3$	2M $ZnSO_4$	0.4-1.4	300 (0.05 A/g)	<25%/100 @ 1A/g	[3]
$Ag_4V_2O_7$	2M $ZnSO_4$	0.4-1.4	160 (0.1 A/g)	<66%/100 @ 1A/g	[3]
$Ag_2V_4O_{11}$	3M $Zn(CF_3SO_3)_2$	0.4-1.7	210 (0.1A/g)	93%/6000 @ 5A/g	[4]
$Ag_{0.33}V_2O_5@V_2O_5$	3M $Zn(CF_3SO_3)_2$	0.2-1.8	312 (0.5A/g)	90%/100 @ 0.5A/g	[5]
β-$AgVO_3$	1.5M $ZnSO_4$	0.4-1.3	283 (0.1 A/g)	65%/200 @0.1A/g	[6]
CuV_2O_6	3M $Zn(CF_3SO_3)_2$	0.3-1.6	427 (0.1A/g)	99%/3000 @ 5A/g	[7]
$Cu_{0.95}V_2O_5$	3M $Zn(CF_3SO_3)_2$	0.2-1.6	405 (0.1A/g)	75%/100 @0.5A/g	[8]
$Cu_{0.34}V_2O_5$	6M $ZnSO_4$	0.25-1.8	315 (0.02A/g)	91%/1000 @ 0.8A/g	[9]
$Cu_3(OH)_2V_2O_7$	3M $ZnSO_4$	0.4-1.4	336 (1A/g)	~100%/3000 @10A/g	[10]
$Cu_3(OH)_2V_2O_7$	2.5M $Zn(CF_3SO_3)_2$	0.2-1.6	216 (0.1A/g)	89%/500 @0.5A/g	[11]
$Cu_xV_2O_5$	2M $ZnSO_4$	0.3-1.4	300 (2A/g)	88%/10000 @ 10A/g	[12]

Table S1: Metrics for Ag or Cu-Vanadium oxide cathodes in Aqueous Zinc Ion Batteries.

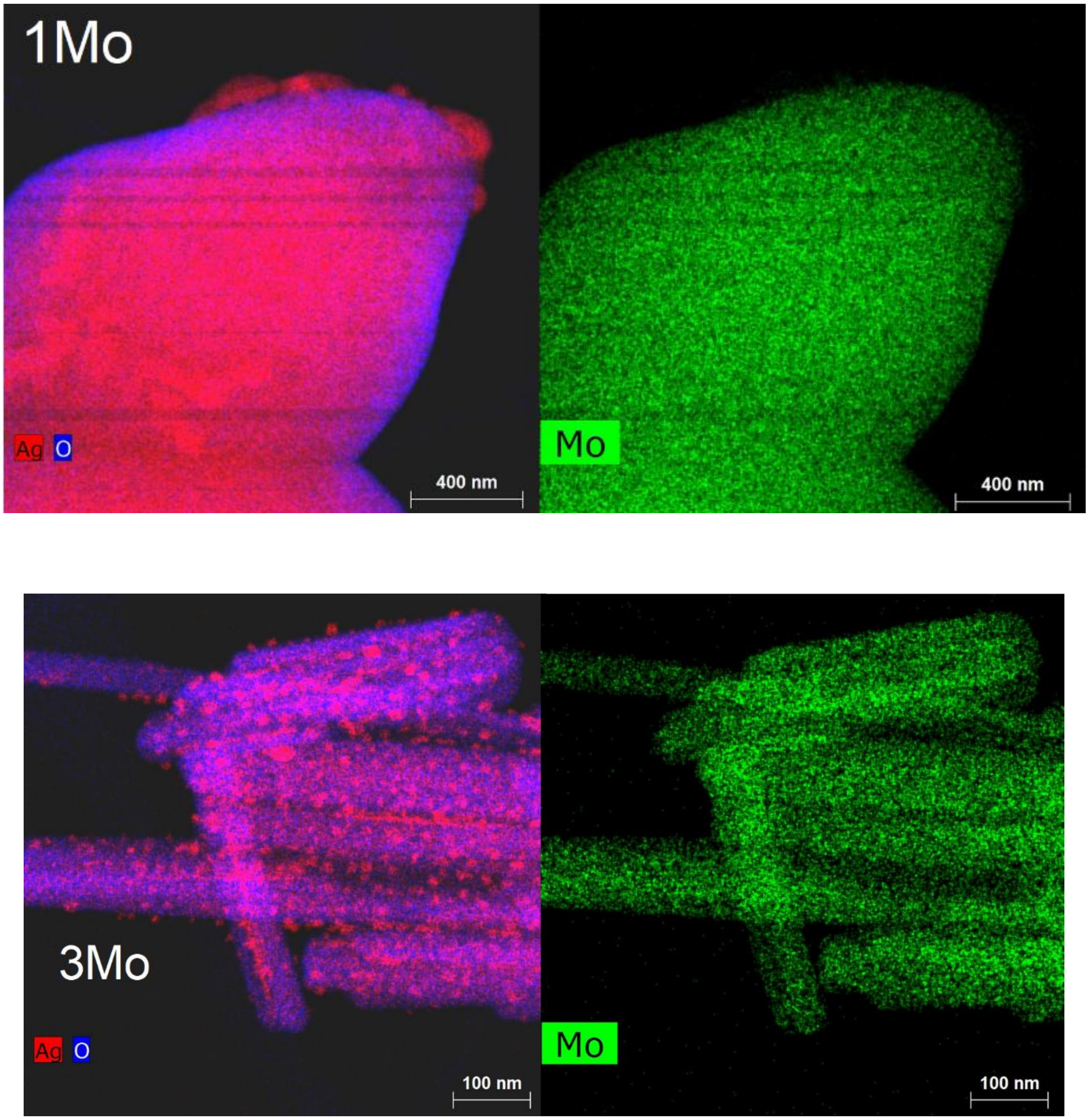

Figure S1: EDS evidence for Ag^{+} autoreduction in the TEM beam. In **1Mo** and **3Mo** the surface particles/protrusions are clearly resolved as pure Ag.

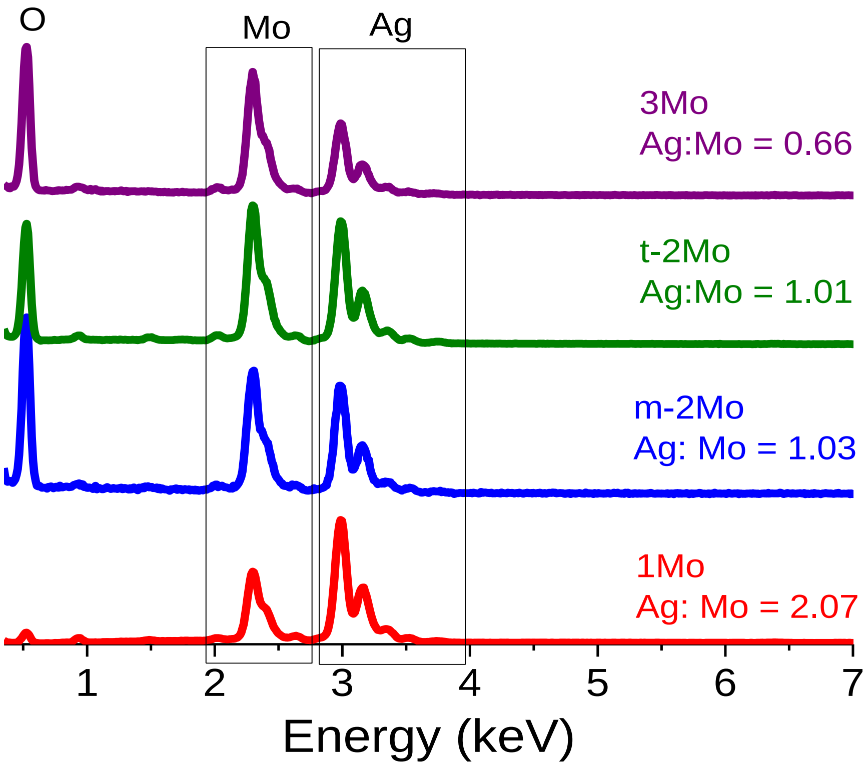

Figure S2: EDS spectra for the HAADF images in the manuscript's Figure 1.

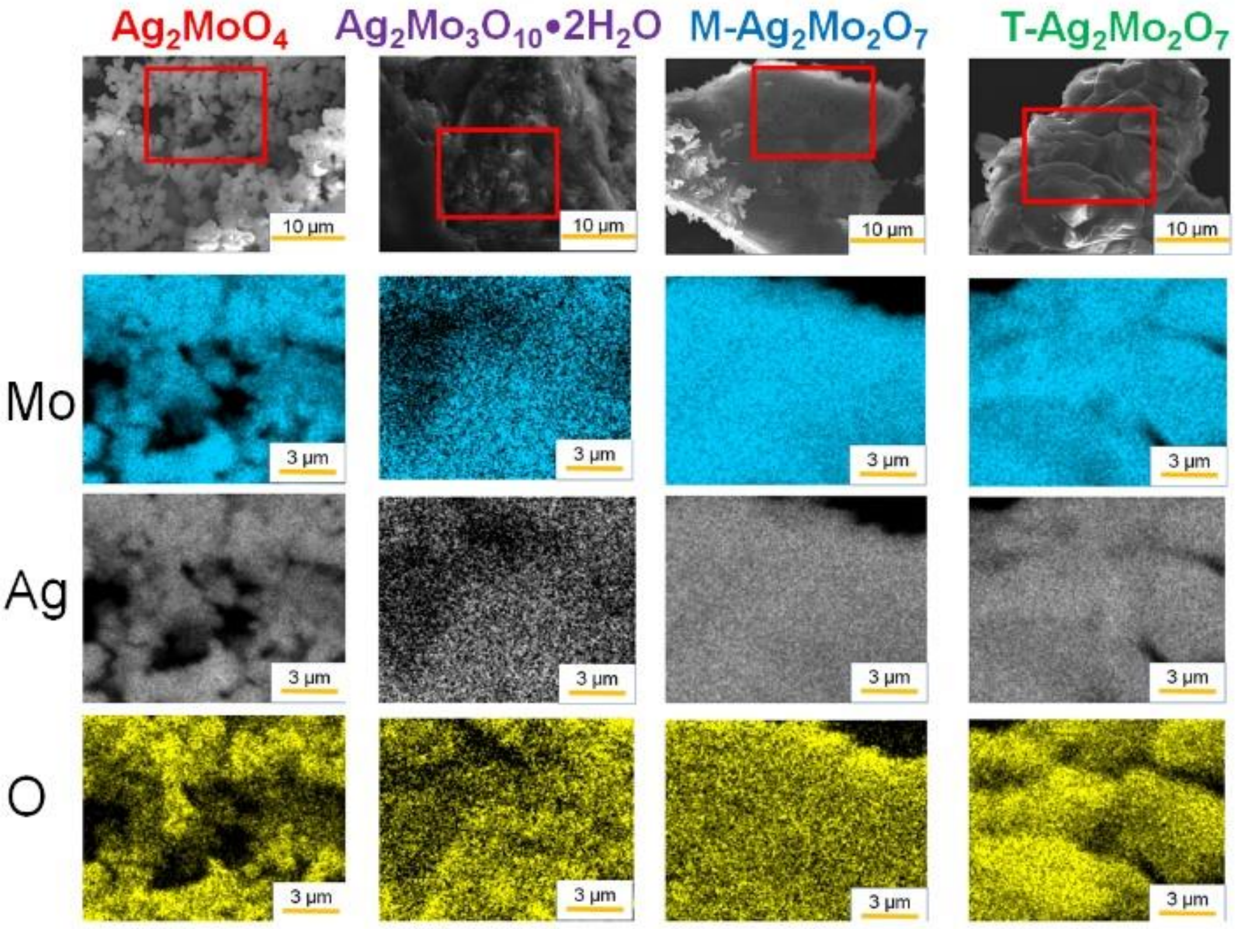

Figure S3. SEM images at 3000x magnification; the red box indicates the region analyzed by EDS mapping.

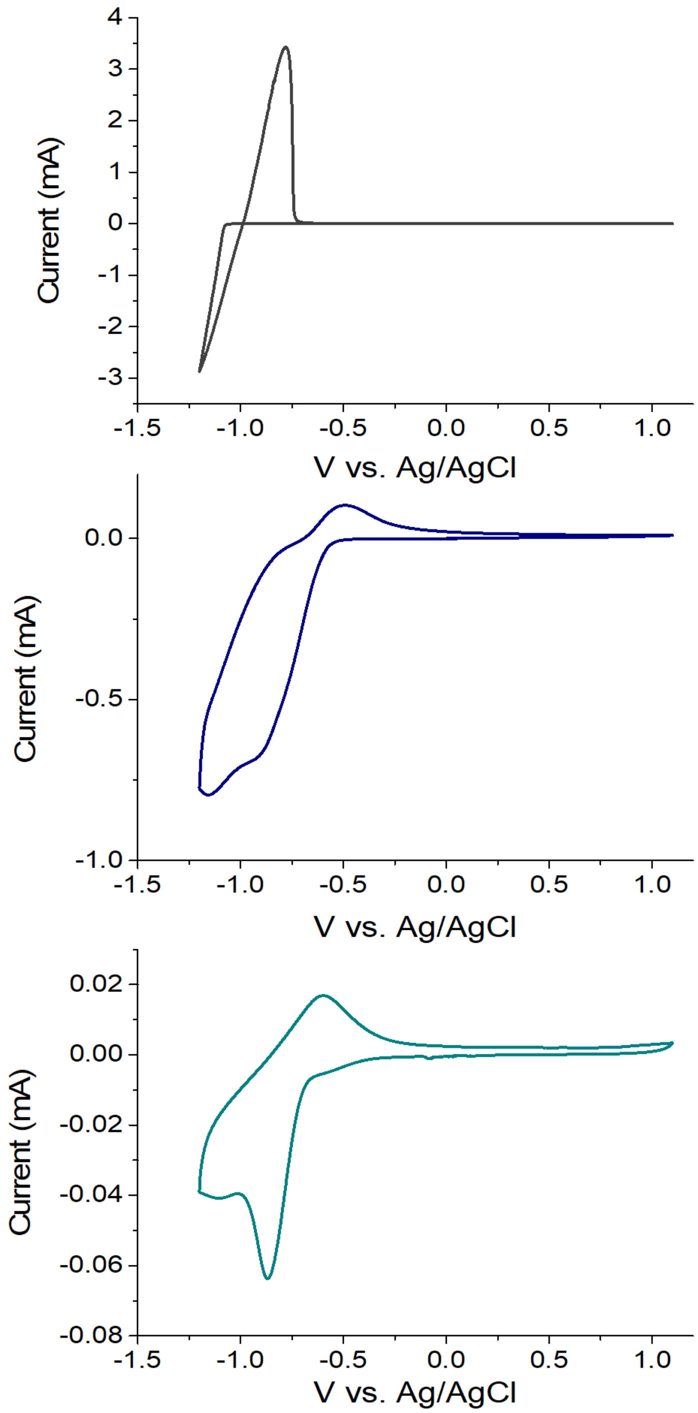

Fig S4: Cyclic voltammograms (20 mV/s) at a glassy carbon electrode of aqueous (top, grey) $ZnSO_4$, (middle, blue) $(NH_4)_2Mo_2O_7$ and (bottom, cyan) Na_2MoO_4.

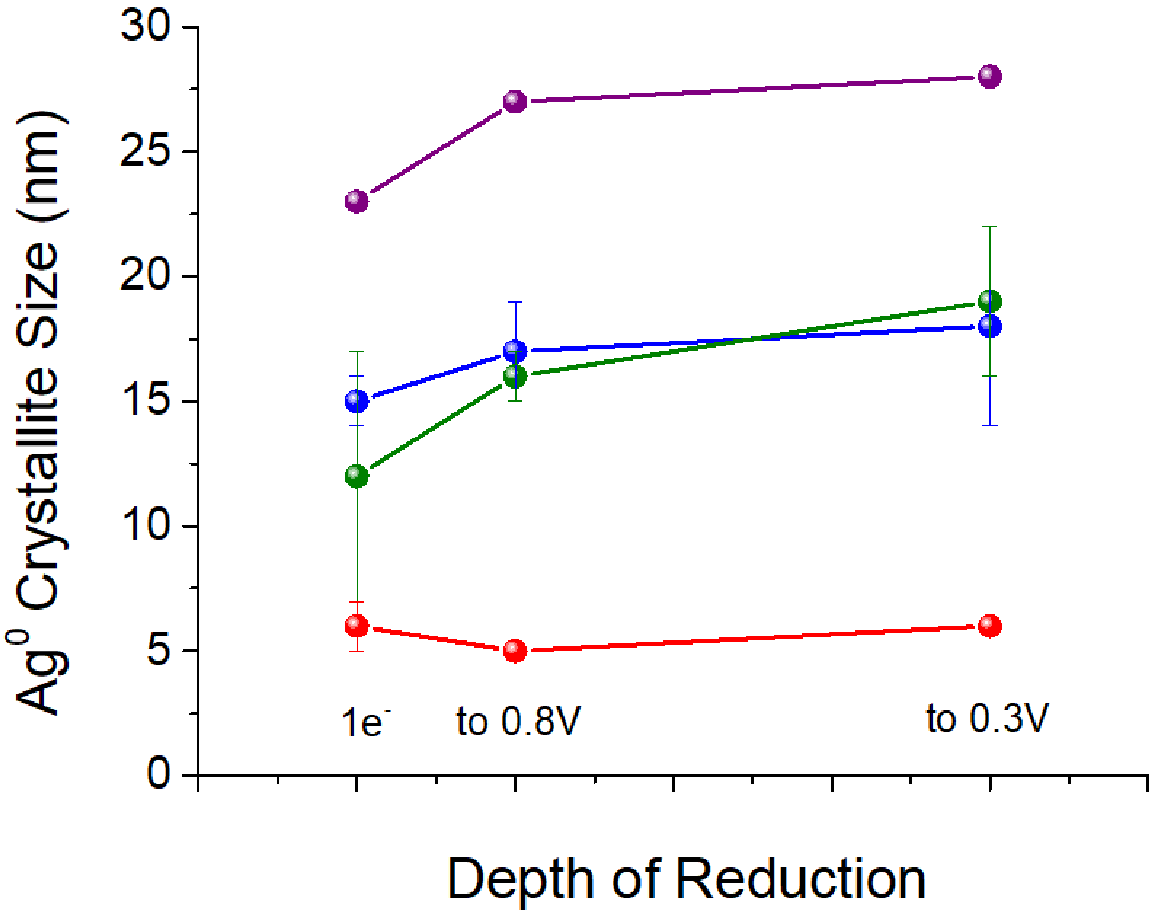

Fig S5: The Scherrer equation determines the Ag crystallite size for each SMO as a function of its reduction. Red: **1Mo**, Blue: **m-2Mo**, Green: **t-2Mo**, Purple: **3Mo**.

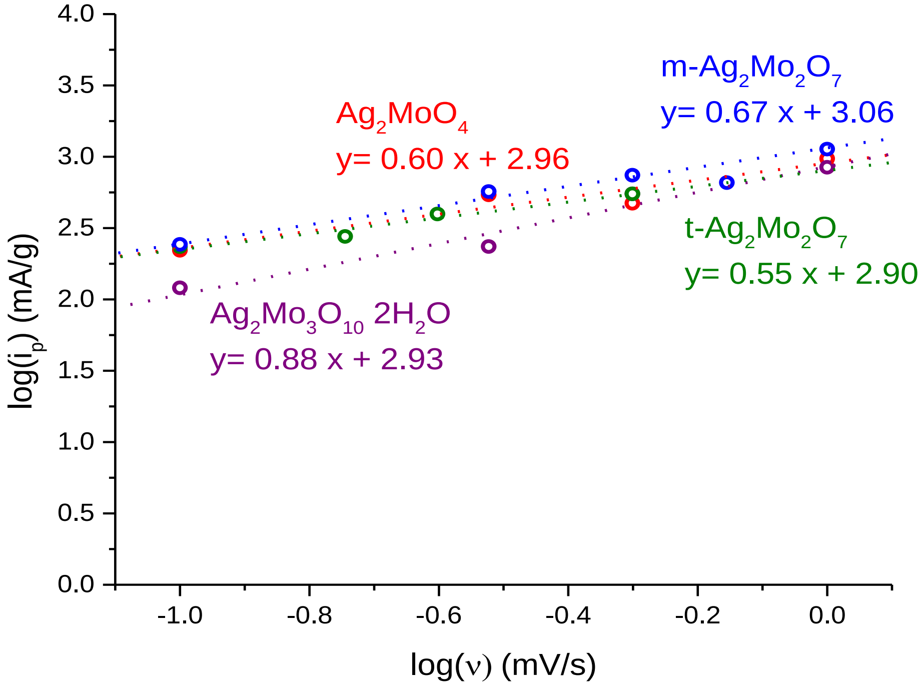

Fig S6: A plot of log(i) vs. log(ν) from variable rate CV's yield a line with slope b between 0.5 (pure diffusion) and 1 (pure capacitance). Here we plot the peak current for the reduction of silver in each SMO.

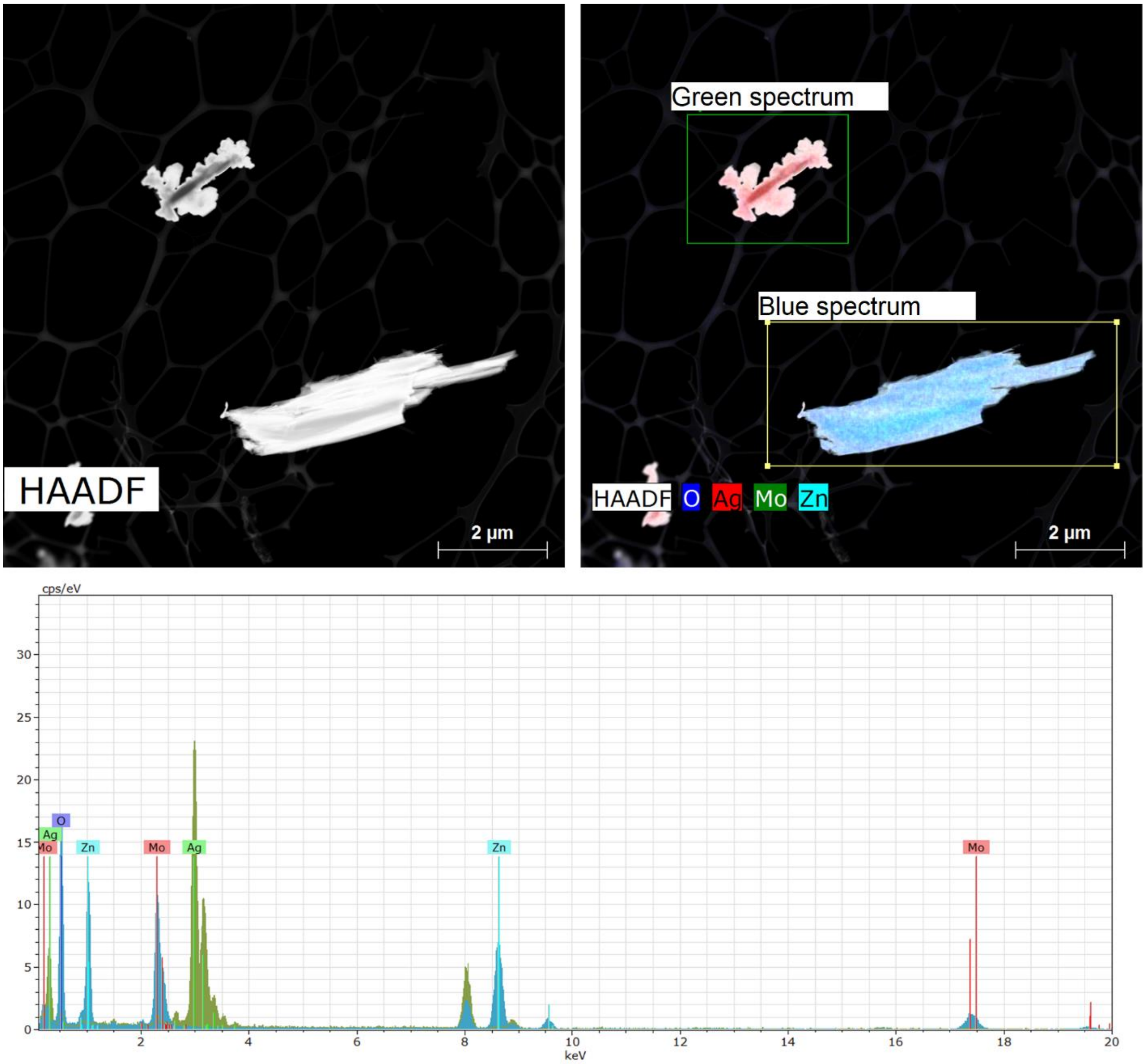

Figure S7: A HAADF/STEM image with EDS data from a 0.8V reduced **m-2Mo** electrode.

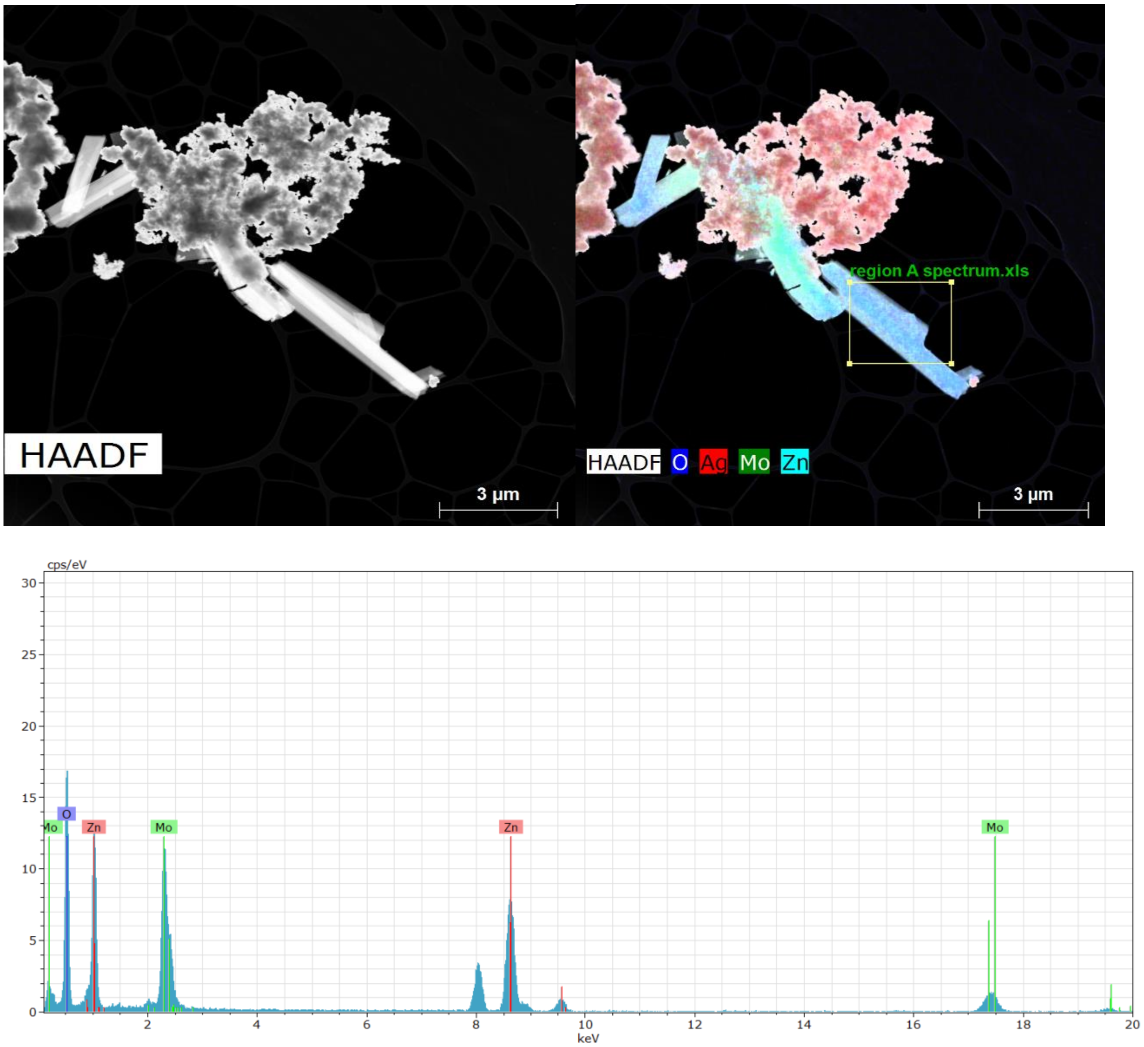

Figure S8: A HAADF/STEM image with EDS data from a 0.8V reduced **t-2Mo** electrode. The spectrum provided is for the region in the yellow box.

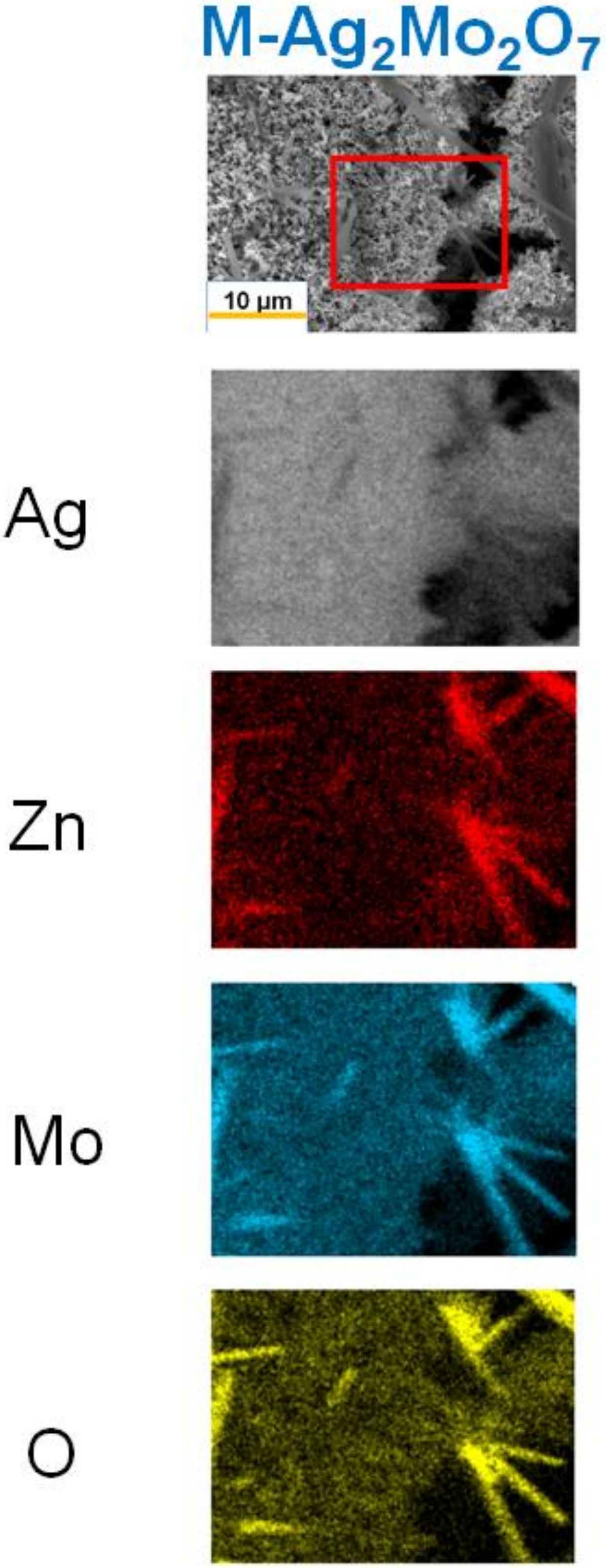

Figure S9: SEM/EDS characterization of 0.8V- reduced **m-2Mo.**

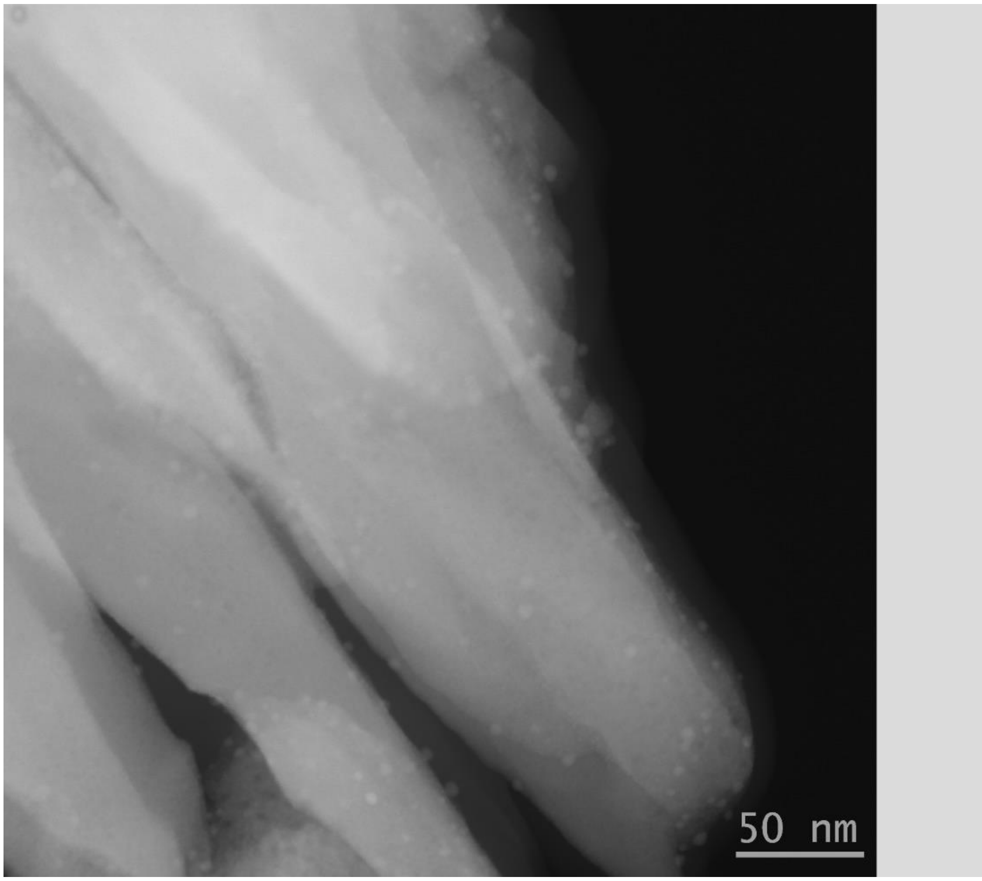

Figure S10: Zoomed in view of a **1Mo** particle reduced to 0.8V.

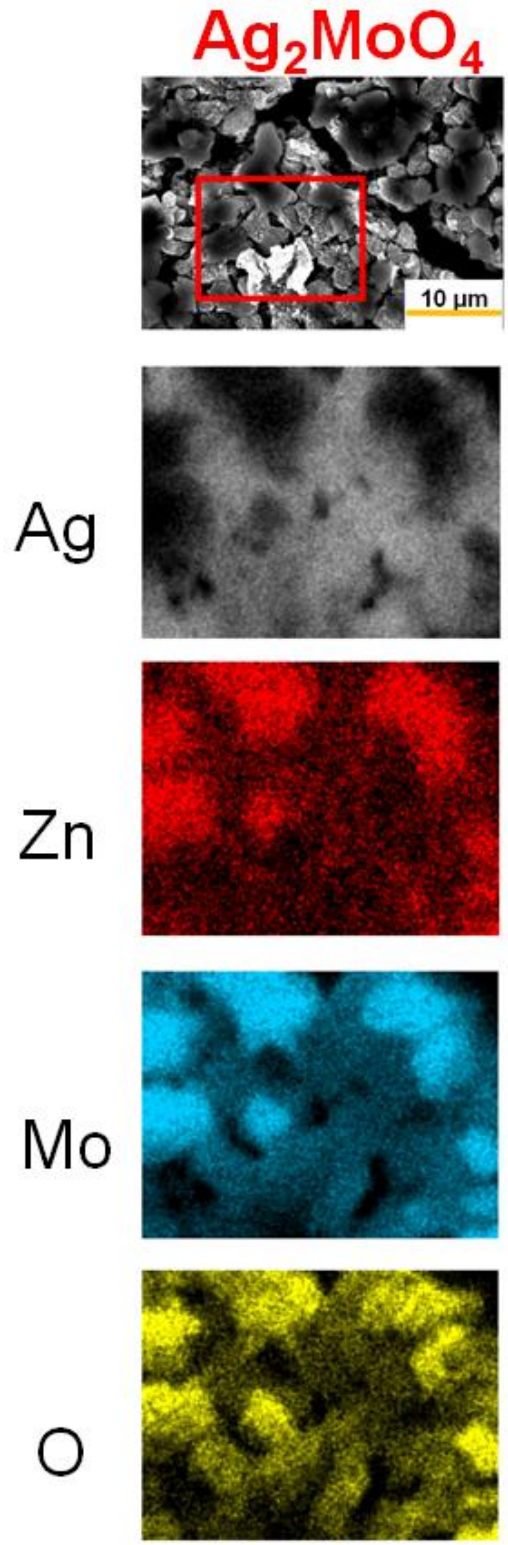

Figure S11: SEM/EDS characterization of 0.8V- reduced **1Mo**.

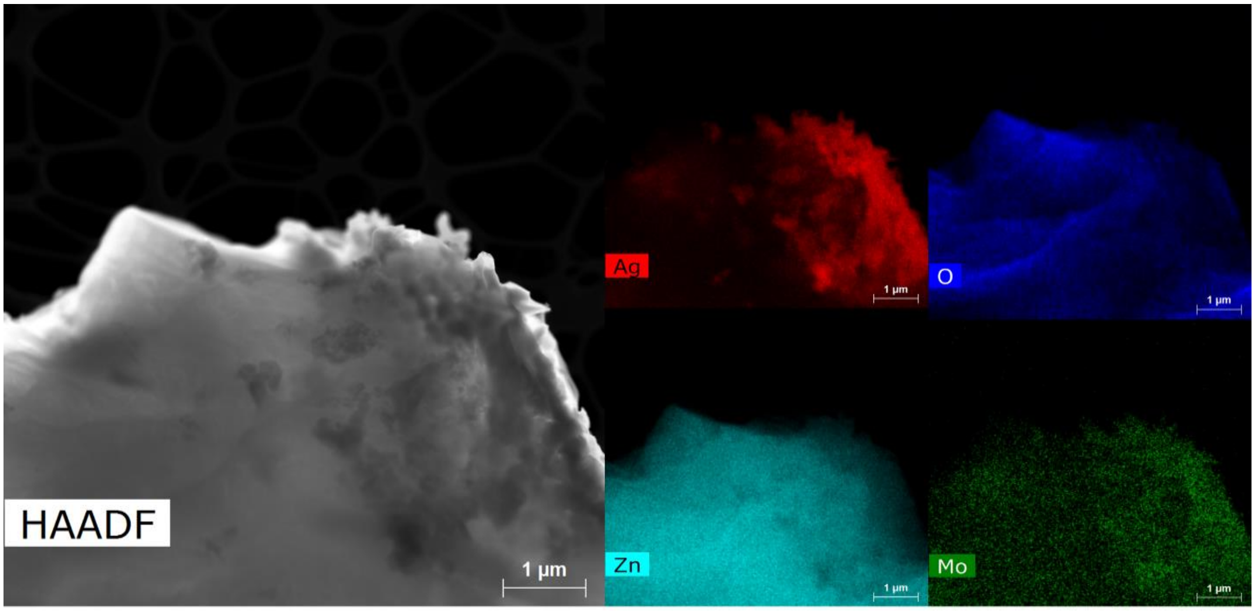

Figure S12: An example of a Zn-O rich particle detected by HAADF/EDS from a 0.8V-reduced **3Mo** electrode; Zn and O represent 74% of the sample shown.

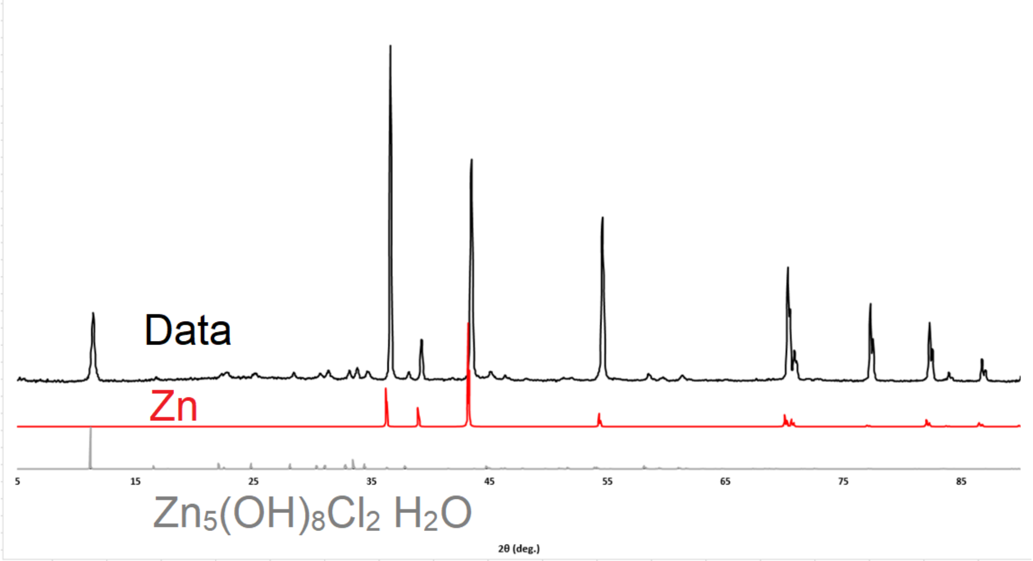

Figure S13: PXRD data of the anode following discharge of **3Mo** in 2M $ZnCl_2$ electrolyte.

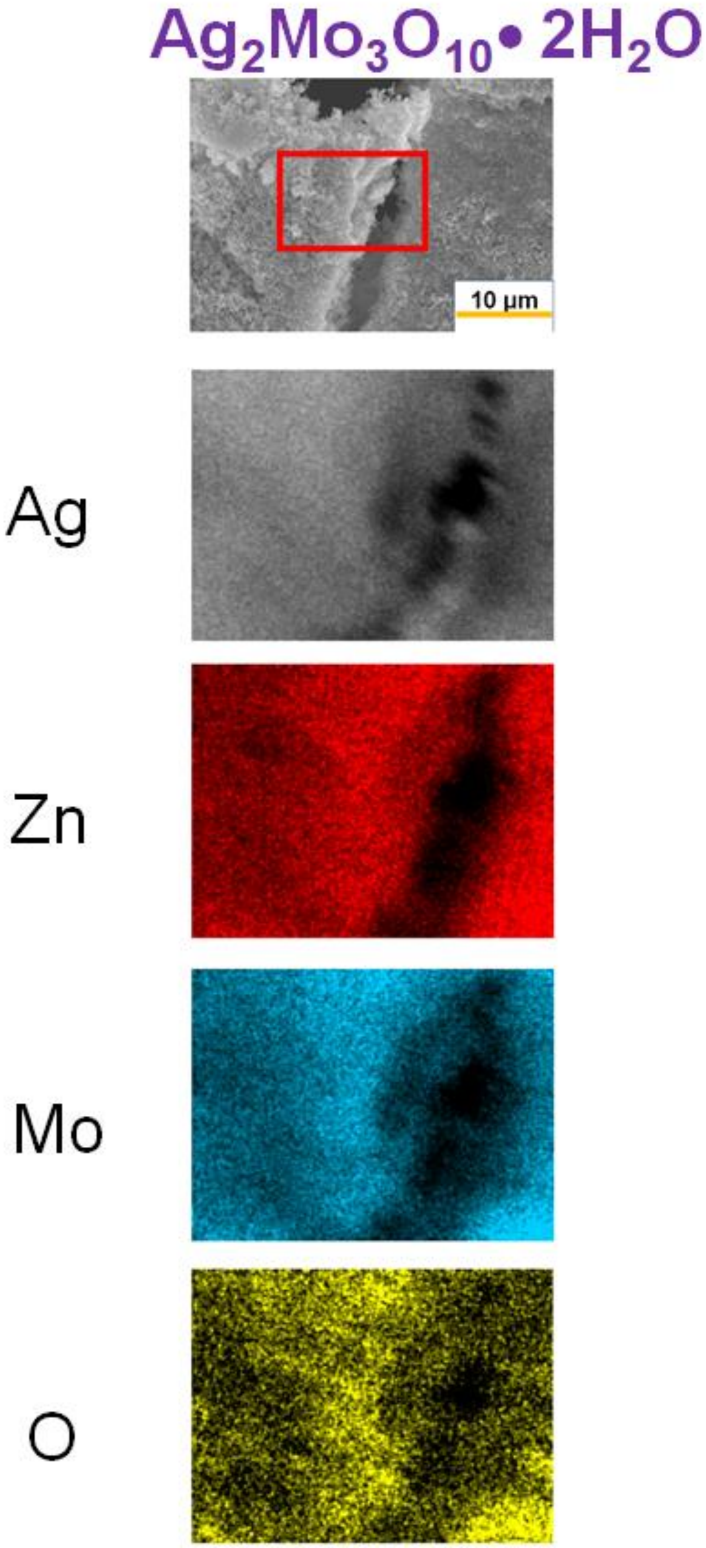

Figure S14: SEM/EDS characterization of 0.8V- reduced **3Mo**. This image clearly contrasts with Figures S9 and S11.

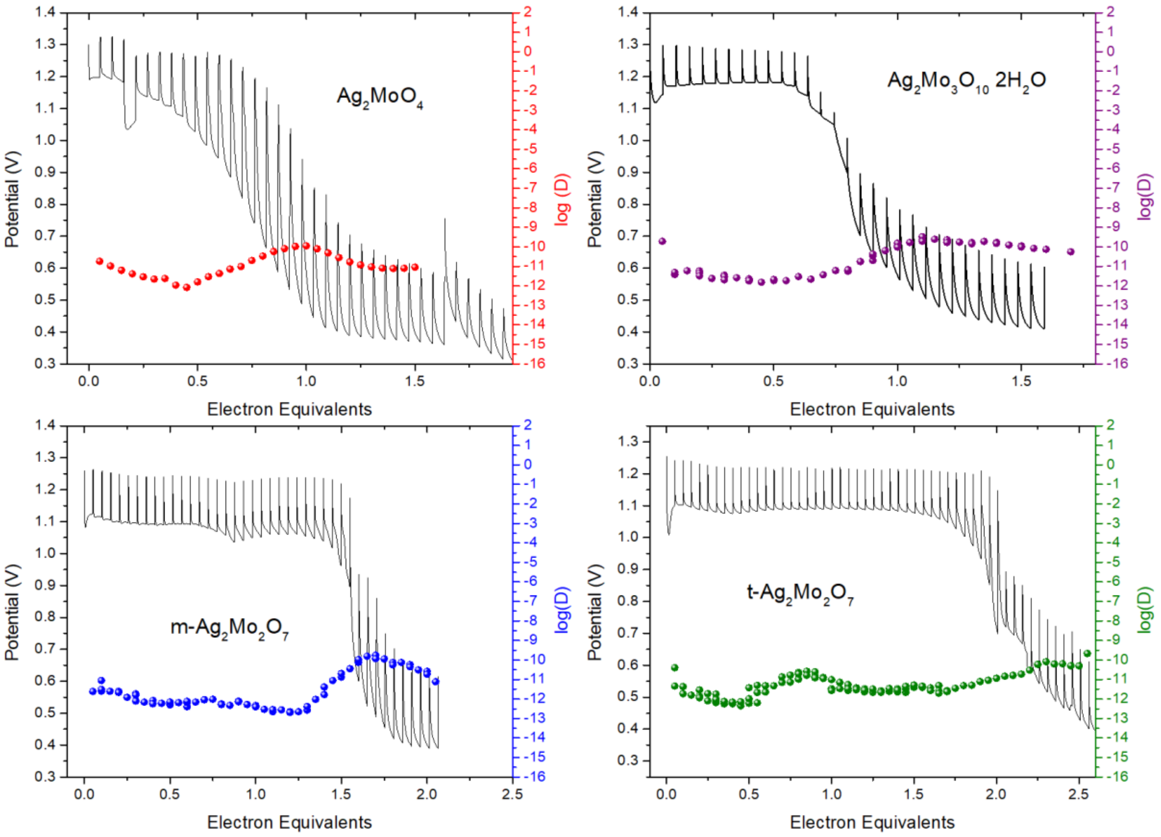

Figure S15. Black traces: Representative data following relaxation after 0.5e$^-$/hr pulses (black traces). Colored circles: Depict 10-pt average smoothed data for log(D) for three replicates.

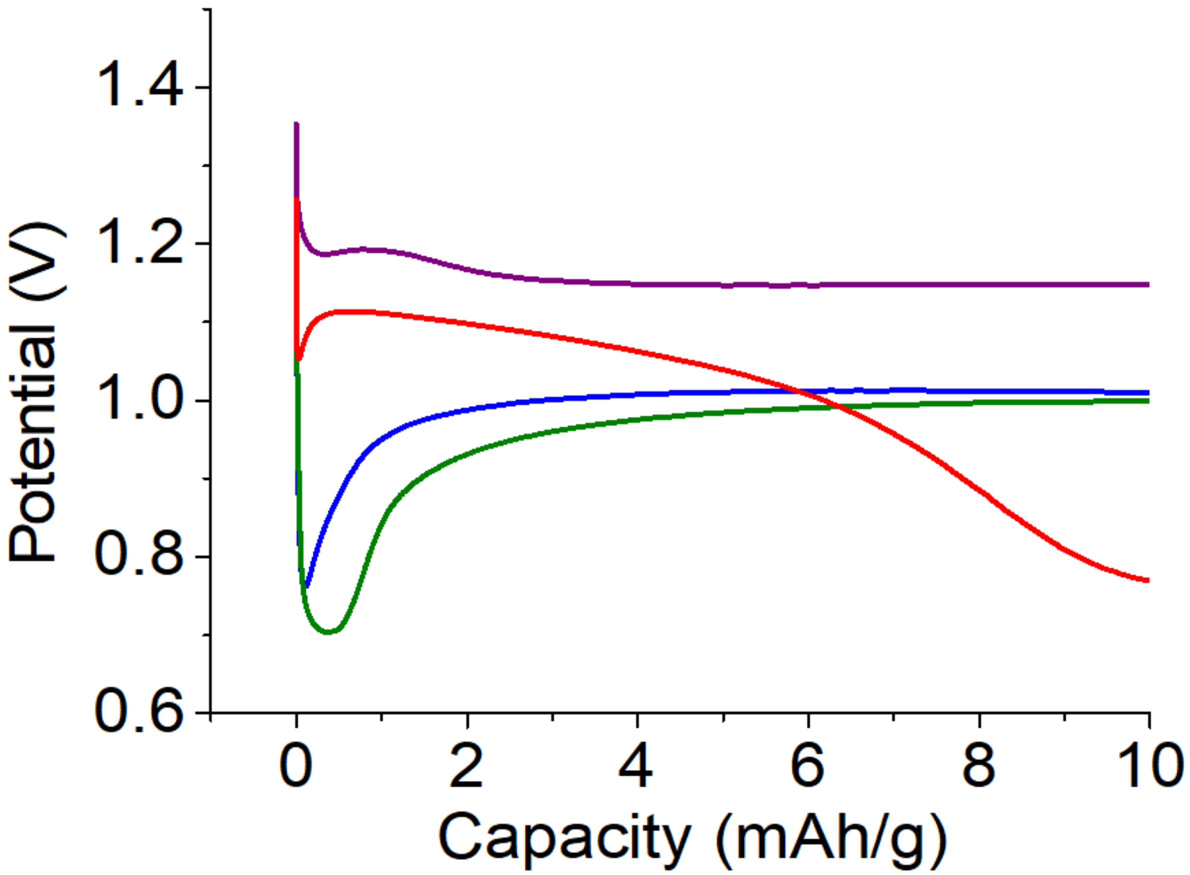

Fig S16: Galvanostatic discharge at 40 mA/g for all SMO's. Purple: **3Mo**, Red: **1Mo**, Blue: **m-2Mo**, Green: **t-2Mo**. The voltage recovery for t-2Mo is clearly slower than its compositional analog **m-2Mo**.

Discussion of 3Mo synthesis:

Hexagonal MoO_3 represents a class of materials with the formula $M_xH_yMoO_{3-\delta}$, where M can be a variety of monovalent cations.[13–15] The 120°C hydrothermal reaction of Na_2MoO_4 and 3 eq. CH_3NH_3Cl (which forms $(CH_3NH_3)_2Mo_7O_{22}$) reported by Dessapt et. al.[16] is considerably time dependent in large part due to hexagonal impurity. If the reaction proceeds longer than 6 hours, hexagonal MoO_3 becomes the predominant phase; we note that we are not the first to observe hexagonal MoO_3 as a cocrystallized impurity in a synthesis of $(CH_3NH_3)_2Mo_7O_{22}$.[17] If the reaction proceeds shorter than 5 hours, formation of $(CH_3NH_3)_2Mo_7O_{22}$ is incomplete. While we are not sure of the identity of this intermediate phase at this time, we found it reacted with Ag^+ under acidic conditions to yield phase-pure hexagonal MoO_3. Hence, the synthesis of $(CH_3NH_3)_2Mo_7O_{22}$ potentially can yield either hexagonal MoO_3 directly or an intermediate which forms hexagonal MoO_3 in the second step (reaction with silver). It becomes crucial to isolate $(CH_3NH_3)_2Mo_7O_{22}$ after it is fully formed and before it further reacts to become hexagonal-MoO_3.

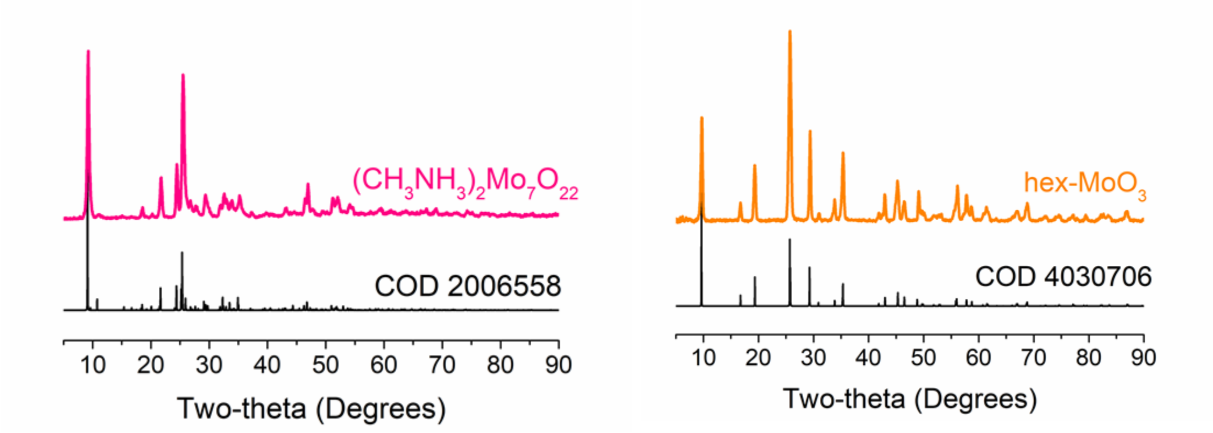

The hexagonal phase is most stabilized in strongly acidic conditions. Therefore, pH alleviates this issue significantly: we found if the reaction occurs at pH 1.5, the hexagonal impurity is avoided more efficiently than at pH 1. Notably reaction of $(CH_3NH_3)_2Mo_7O_{22}$ with Ag^+ at pH>1 formed the kinetically favorable m-$Ag_2Mo_2O_7$, so changes in pH in the second step are not possible. Rather, our modifications reflected in the Experimental section best avoid the hexagonal impurity by reacting $(CH_3NH_3)_2Mo_7O_{22}$ with a small excess of Ag^+ at pH 1, and with longer reaction times (>6 hr). This excess favors the formation of the more silver-rich $Ag_2Mo_3O_{10}$ over $Ag_{0.16}H_xMoO_3$, and the low pH avoids thermodynamically favorable triclinic $Ag_2Mo_2O_7$.

Pure $(CH_3NH_3)_2Mo_7O_{22}$ obtains a blue tint in direct light and following XRD measurement.[18] We found the use of blue tinted $(CH_3NH_3)_2Mo_7O_{22}$ in the second step does not change the phase purity of the $Ag_2Mo_3O_{10}$•$2H_2O$ product and we believe any XRD- reduced Mo dissolves in the acidic conditions. Nonetheless, for this manuscript we only report data on $Ag_2Mo_3O_{10}$•$2H_2O$ synthesized using $(CH_3NH_3)_2Mo_7O_{22}$ sample portions that were not x-rayed directly.

(1) Lan, B.; Peng, Z.; Chen, L.; Tang, C.; Dong, S.; Chen, C.; Zhou, M.; Chen, C.; An, Q.; Luo, P. *J. Alloys Compd.* **2019**, *787*, 9–16.

(2) Shan, L.; Yang, Y.; Zhang, W.; Chen, H.; Fang, G.; Zhou, J.; Liang, S. *Energy Storage Mater.* **2019**, *18*, 10–14.

(3) Guo, S.; Fang, G.; Liang, S.; Chen, M.; Wu, X.; Zhou, J. *Acta Mater.* **2019**, *180*, 51–59.

(4) Li, Q.; Liu, Y.; Ma, K.; Yang, G.; Wang, C. *Small Methods* **2019**, *3*, 1900637.

(5) Zeng, J.; Chao, K.; Wang, W.; Wei, X.; Liu, C.; Peng, H.; Zhang, Z.; Guo, X.; Li, G. *Inorg. Chem. Front.* **2019**, *6*, 2339–2348.

(6) Liu, H.; Wang, J.; Sun, H.; Li, Y.; Yang, J.; Wei, C.; Kang, F. *J. Colloid Interface Sci.* **2020**, *560*, 659–666.

(7) Liu, Y.; Li, Q.; Ma, K.; Yang, G.; Wang, C. *ACS Nano* **2019**, *13*, 12081–12089.

(8) Yu, X.; Hu, F.; Guo, Z.-Q. Q.; Liu, L.; Song, G.-H. H.; Zhu, K. *Rare Met.* **2021**.

(9) Chae, M. S.; Attias, R.; Dlugatch, B.; Gofer, Y.; Aurbach, D. *ACS Appl. Energy Mater.* **2021**, *4*, 10197–10202.

(10) Shan, L.; Zhou, J.; Han, M.; Fang, G.; Cao, X.; Wu, X.; Liang, S. *J. Mater. Chem. A* **2019**, *7*, 7355–7359.

(11) Chen, L.; Yang, Z.; Wu, J.; Chen, H.; Meng, J. *Electrochim. Acta* **2020**, *330*, 135347.

(12) Yang, Y.; Tang, Y.; Liang, S.; Wu, Z.; Fang, G.; Cao, X.; Wang, C.; Lin, T.; Pan, A.; Zhou, J. *Nano Energy* **2019**, *61*, 617–625.

(13) McCarron, E. .; Thomas, D. M.; Calabrese, J. C. *Inorg. Chem.* **1987**, *26*, 370–373.

(14) Guo, J.; Zavalij, P.; Whittingham, M. S. *J. Solid State Chem.* **1995**, *117*, 323–332.

(15) Lunk, H.; Hartl, H.; Hartl, M. A.; Fait, M. J. G.; Shenderovich, I. G.; Feist, M.; Frisk, T. A.; Daemen, L. L.; Mauder, D.; Eckelt, R.; Gurinov, A. A. *Inorg. Chem.* **2010**, *49*, 9400–9408.

(16) Hakouk, K.; Deniard, P.; Lajaunie, L.; Guillot-Deudon, C.; Harel, S.; Wang, Z.; Huang, B.; Koo, H.-J.; Whangbo, M.-H.; Jobic, S.; Dessapt, R. *Inorg. Chem.* **2013**, *52*, 6440–6449.

(17) Cui, X.; Yu, S. H.; Li, L.; Biao, L.; Li, H.; Mo, M.; Liu, X. M. *Chem. - A Eur. J.* **2004**, *10*, 218–223.

(18) Zavalij, P. Y.; Whittingham, M. S. *Acta Crystallogr. Sect. C Cryst. Struct. Commun.* **1997**, *53*, 1374–1376.
